# Proteins Involved in Platelet Signaling Are Differentially Regulated in Acute Coronary Syndrome: A Proteomic Study

**DOI:** 10.1371/journal.pone.0013404

**Published:** 2010-10-14

**Authors:** Andrés Fernández Parguiña, Lilian Grigorian-Shamajian, Rosa M. Agra, Elvis Teijeira-Fernández, Isaac Rosa, Jana Alonso, Juan E. Viñuela-Roldán, Ana Seoane, José Ramón González-Juanatey, Ángel García

**Affiliations:** 1 Departamento de Farmacoloxía, Facultade de Farmacia, Universidade de Santiago de Compostela, Santiago de Compostela, Spain; 2 Servicio de Cardiología y Unidad Coronaria, Hospital Clínico Universitario de Santiago, Santiago de Compostela, Spain; 3 Servicio de Cardiología, Complejo Hospitalario Universitario de Vigo, Vigo, Spain; 4 Laboratorio de Proteómica, Instituto de Investigaciones Sanitarias, Hospital Clínico Universitario, Santiago de Compostela, Spain; 5 Laboratorio de Inmunología, Hospital Clínico Universitario, Santiago de Compostela, Spain; Universität Würzburg, Germany

## Abstract

**Background:**

Platelets play a fundamental role in pathological events underlying acute coronary syndrome (ACS). Because platelets do not have a nucleus, proteomics constitutes an optimal approach to follow platelet molecular events associated with the onset of the acute episode.

**Methodology/Principal Findings:**

We performed the first high-resolution two-dimensional gel electrophoresis-based proteome analysis of circulating platelets from patients with non-ST segment elevation ACS (NSTE-ACS). Proteins were identified by mass spectrometry and validations were by western blotting. Forty protein features (corresponding to 22 unique genes) were found to be differentially regulated between NSTE-ACS patients and matched controls with chronic ischemic cardiopathy. The number of differences decreased at day 5 (28) and 6 months after the acute event (5). Interestingly, a systems biology approach demonstrated that 16 of the 22 differentially regulated proteins identified are interconnected as part of a common network related to cell assembly and organization and cell morphology, processes very related to platelet activation. Indeed, 14 of those proteins are either signaling or cytoskeletal, and nine of them are known to participate in platelet activation by αIIbβ3 and/or GPVI receptors. Several of the proteins identified participate in platelet activation through post-translational modifications, as shown here for ILK, Src and Talin. Interestingly, the platelet-secreted glycoprotein SPARC was down-regulated in NSTE-ACS patients compared to stable controls, which is consistent with a secretion process from activated platelets.

**Conclusions/Significance:**

The present study provides novel information on platelet proteome changes associated with platelet activation in NSTE-ACS, highlighting the presence of proteins involved in platelet signaling. This investigation paves the way for future studies in the search for novel platelet-related biomarkers and drug targets in ACS.

## Introduction

Platelets play a fundamental role in the initiation, development and total extent of myocardial damage in acute coronary syndrome (ACS). The rupture of an unstable atherosclerotic plaque leads to the local generation of thrombin and deposition of fibrin. This in turn promotes platelet activation, adhesion and aggregation, and the formation of intra-coronary thrombus [1], [2]. Patients with complete occlusion may manifest ST segment elevation myocardial infarction (STEMI) if the lesion occludes an artery supplying a substantial volume of myocardium, but the same occlusion in the presence of extensive collateralization may manifest as a non-ST segment elevation acute coronary syndrome (NSTE-ACS) [3].

In recent years proteomics has emerged as a novel technology applied to cardiovascular research with the aim of identifying novel biomarkers that could help to improve the diagnosis and illuminate disease mechanisms [4], [5]. In the context of ACS, proteomics has been primarily applied to the study of plasma [6] and monocytes [7] from ACS patients, leading to the identification of some potential biomarkers. However, to the best of our knowledge this is the first time high-resolution proteomics is applied to the study of platelets in ACS.

Because platelets do not have a nucleus, proteomics is a perfect tool to approach their biochemistry. In the last ten years proteomics has been successfully applied to platelet research [8], [9]. We have been among the pioneers in the field, with a primary focus on signaling studies [10]–[13]. Based on our previous experience, we carried out a high-resolution two-dimensional gel electrophoresis (2-DE)-based proteome analysis of platelets from NSTE-ACS patients versus stable coronary artery disease (SCAD) controls. The objective was to identify, through a global proteomic approach, differentially regulated platelet proteins that could provide information on the molecular mechanisms playing a major role in the unwanted platelet activation associated with the ACS.

## Materials and Methods

### Ethics Statement

The study was approved by the local Ethics Committee (Galician Clinical Investigation Ethics Committee) and developed according to the principles outlined in the Declaration of Helsinki. All patients included in the study signed a written informed consent.

### Patients

Eighteen patients admitted into a tertiary hospital from the northwest of Spain presenting with NSTE-ACS, defined as angina pain of at least 10 min duration with troponin elevation and/or ST-T changes in the electrocardiogram, entered the study. Exclusion criteria were inflammatory or neoplastic diseases, coagulation disorders, platelet-associated disorders, other significant heart disease except left ventricular hypertrophy secondary to hypertension, chronic drug therapy (except for drugs required to treat pre-existing clinical atherosclerosis or its risk factors), and having suffered from surgical procedures, major traumatisms, thromboembolic events or revascularization procedures in the previous 3 months.

At the moment of diagnosis, the patients were asked to participate in the study. In case of acceptance, they signed the informed consent, and 27 mL of blood were collected in sodium citrate BD Vacutainer tubes for analysis. All samples were obtained within the first 24 hours following the initiation of the symptoms and immediately after arrival at the emergency department. In most cases, patients were administered aspirin before arrival to hospital, so aspirin-pretreated patients were admitted in the study. Some patients had also clopidogrel treatment and were also admitted. However, patients previously treated with anti-αIIbβ3 drugs were excluded. A second blood sample was taken on day 5 to investigate if there was a fast reversion of the changes observed in the platelet proteome on admission. Another blood sample was taken after 6 months, as we hypothesized that most changes observed on admission could be normalized after that time since the status of the ACS patients should be then closer to that of chronic controls. Blood was also collected from 10 matched stable coronary artery disease patients (SCAD), suffering from stable chronic ischemic cardiopathy. Such a group was decided to be the most adequate control group due to the clinical characteristics of the ACS patients. SCAD patients were required not to have a history of acute cardiovascular event within the year before the inclusion in the study.

### Platelet isolation

Platelets were isolated by an established method that limits contamination from other blood cells as described previously [14]. Washed platelets were resuspended in Tyrodes-HEPES (134 mM NaCl, 0.34 mM Na_2_HPO_4_, 2.9 mM KCl, 12 mM NaHCO_3_, 20 mM HEPES, 5 mM glucose, 1 mM MgCl_2_, pH 7.3) at 5×10^8^ platelets/ml, and incubated at room temperature for 30 min (resting step). Five minutes before the end of the resting step, EGTA was added to a final concentration of 1 mM to prevent spontaneous aggregation during centrifugation [14]. Platelets were then spun down at 10,000 g and, after addition of 5 µL of a protease inhibitor cocktail (Sigma, St. Louis, MO, USA), immediately frozen in liquid nitrogen for a few seconds followed by the addition of 2-DE sample buffer (see below). Protein samples were stored at −80°C.

### Surface expression of platelet αIIbβ3

The determination of the platelet surface expression of αIIbβ3 integrin was performed by flow cytometry using platelet-rich plasma (PRP) aliquots and specific antibodies as indicated in Supporting Information S1.

### Two-dimensional gel electrophoresis

Protein quantitation was done with the *Coomassie plus protein reagent* (Thermo Scientific, Asheville, NC). The amount of platelet protein obtained from each patient was of 1450.6±401.5 µg. Six-hundred micrograms of protein were loaded onto each gel to allow detection of low abundant proteins. The remaining protein was used for western blotting and, when possible, preserved for proteomics in case of unexpected technical problems (e.g. gel breakage). Therefore, regarding the proteomic study, an individual gel was run for each blood sample, from each patient, at three different times: on admission, 5 days, and 6 months.

For each sample, protein was dissolved in 500 µl of 2D sample buffer (5 M urea, 2 M thiourea, 2 mM tributyl-phosphine, 65 mM DTT, 65 mM CHAPS, 0.15 M NDSB-256, 1 mM sodium vanadate, 0.1 mM sodium fluoride, and 1 mM benzamidine). Ampholytes (Servalyte 4–7) were added to the sample to a final concentration of 1.6% (v/v). First dimension was on immobilized pH gradient (IPG) strips 4–7, 24 cm (GE Healthcare). Second dimension was by SDS-polyacrylamide gel electrophoresis (PAGE) on 10% gels. Further information on the 2-DE protocol can be found in Supporting Information S1.

Following electrophoresis, gels were fixed in 10% methanol/7% acetic acid for 1 hour, and stained overnight with Sypro Ruby fluorescent dye. After staining, gels were washed for 1 hour in 10% methanol/7% acetic acid, and scanned in a Typhoon 9410 (GE Healthcare).

### Differential image analysis

Due to the high amount of images to be processed, images were sent to the Ludesi Analysis Center (Lund, Sweden, http://www.ludesi.com) for professional image analysis using Ludesi REDFIN 3 software. That allowed an optimal control over potential technical variations. Spot detection, segmentation and matching followed a strict protocol to ensure a high level of correctness. Gels were matched by using all-to-all spot matching, avoiding the bias caused by the use of a reference gel. The integrated intensity of each of the spots was measured, and the background corrected and normalized. Normalization removes systematic gel intensity differences originating, for example, from variations in staining, scanning time and protein loading by mathematically minimizing the median expression difference between matched spots. This allows a satisfactory quantification and comparison of different gels. Differential expression of proteins was defined on the basis of >1.5-fold change between group averages and p<0.05.

### Mass spectrometric analysis

Protein features chosen for mass spectrometric analysis were excised from the gels and manually in-gel digested with trypsin following the protocol defined by Shevchenko [15]. Proteins were reduced with DTT and alkylated with iodoacetamide prior to trypsin digestion. For MS analysis, dried peptides were dissolved in 4 µL of 0.5% formic acid. Equal volumes (0.5 µL) of peptide and matrix solution, consisting of 3 mg alpha-cyano-4-hydroxycinnamic acid (α-CHCA) dissolved in 1 mL of 50% acetonitrile in 0.1% trifluoroacetic acid, were deposited using the thin layer method, onto a 384 Opti-TOF MALDI plate (Applied Biosystems). Mass spectrometric data were obtained in an automated analysis loop using 4800 MALDI-TOF/TOF analyzer (Applied Biosystems). MS spectra were acquired in reflectron positive-ion mode with a Nd∶YAG, 355 nm wavelength laser, averaging 1000 laser shots and using at least three trypsin autolysis peaks as internal calibration. All MS/MS spectra were performed by selecting the precursors with a relative resolution of 300 (FWHM) and metastable suppression. Automated analysis of mass data was achieved by using the 4000 Series Explorer Software V3.5. MS and MS/MS spectra data were combined through the GPS Explorer Software v3.6. Database search was performed with the Mascot v2.1 search tool (Matrix Science, London, UK) screening SwissProt (release 56.0). Searches were restricted to human taxonomy allowing carbamidomethyl cysteine as a fixed modification and oxidized methionine as potential variable modification. Both the precursor mass tolerance and the MS/MS tolerance were set at 30 ppm and 0.35 Da, respectively, allowing 1 missed tryptic cleavage site. All spectra and database results were manually inspected in detail using the above software.

### Western blotting

Western blot analyses were carried out on PVDF membranes using the following primary antibodies and dilutions: mouse anti-ILK (1∶200), rabbit anti-Src (1∶200) and rabbit anti-SPARC (1∶500) - all from Santa Cruz Biotechnology, Inc. (Delaware, CA, USA) - and mouse anti-β-actin (1∶1000) from Millipore (Temecula, CA, USA). Membranes were exposed to horseradish peroxidase-labelled goat anti-rabbit or goat anti-mouse antibodies (dilution 1∶2000) (Pierce, Rockford, USA), processed using an enhanced-chemiluminiscence system (ECL, Pierce, Rockford, USA) and quantified by densitometry. Further details on the western blot protocol can be found in Supporting Information S1.

### Ingenuity Pathways Analysis

Ingenuity Pathways Analysis software (Ingenuity Systems, CA) was used to investigate possible interactions between all the identified proteins. Interactive pathways were generated to observe potential direct and indirect relations among the differentially expressed proteins.

### Statistical analysis

Data for categorical or dichotomous variables are expressed as percentages and compared using Chi-squared test or Fisher's exact test. Data for continuous variables are expressed as the mean ± standard deviation and compared by Mann-Whitney test. The differential proteomic analysis was done analyzing all the spots between patients with ACS and SCAD; for a given spot the p-value was calculated using the quantified and normalized volumes for the matched spot in each of the images.

All probability values were 2-tailed, and values of <0.05 were considered to indicate statistical significance. All analyses were performed using SPSS 15.0 software for Windows (SPSS Inc., Tokyo, Japan).

## Results

### Baseline patients' characteristics

Eighteen patients admitted to hospital with the diagnosis of NSTE-ACS and ten age and gender-matched patients with stable coronary artery disease were included in the study. There were only few clinical differences between both groups as shown in Table 1.

**Table 1:** Baseline characteristics of NSTE-ACS and SCAD patients.

Variable	NSTE-ACS (n = 18 patients)	SCAD (n = 10 patients)
Age (years)	66.4±11.8	62.0±13.0
Females (%)	33.3	20.0
Body Mass Index (kg/m^2^)	29.1±3.6	27.0±2.7
Systolic Blood Pressure (mmHg)	139.3±27.6	134.6±15.6
Diastolic Blood Pressure (mmHg)	81.1±18.6	81.8±13.9
Heart Rate (bpm)	73.1±10.5	74.2±15.6
Hx Arterial Hypertension (%)	61.1	50.0
Hx Diabetes Mellitus (%)	22.2	30.0
Hx Smoking (%)	16.7	10.0
Hx Dyslipidemia (%)*	83.3	40.0
Hx Coronary Artery Disease (%)*	27.8	100
Hx Cerebro-vascular Disease (%)	16.7	10.0
Hx Congestive Heart Failure (%)	11.1	0
Hx Peripheral Artery Disease (%)	5.6	20.0
***Laboratory Measurements***		
Hemoglobin (g/dl)	14.3±1.6	13.9±1.3
Leucocites/µL	9748.9±2887.9	8744.3±2030.9
Platelets/µL	228994.4±72644.1	241828.6±108296.5
Mean Platelet Volume (fL)	8.9±1.4	8.6±1.3
Platelets Distribution Width (%)	56.3±8.4	51.3±2.3
Glucose (mg/dl)*	129.0±50.6	97.7±13.2
Creatinin (mg/dl)	0.9±0.2	0.9±0.2
Proteins (mg/dl)	6.6±0.5	6.9±0.4
Cholesterol (mg/dl)	188.4±30.3	151.1±35.9
LDL-Cholesterol (mg/dl)	107.8±36.3	91.6±39.5
HDL-Cholesterol (mg/dl)	32.9±16.5	36.8±6.7
Triglycerides (mg/dl)	180.1±107.5	145.6±107.5
***Chronic Treatments***		
Anticoagulants (%)	5.6	0
ACE Inhibitors (%)	22.2	40.0
Angiotensin Receptor Blockers (%)	22.2	20.0
Statins (%)*	55.6	100
Aspirin (%)* a	22.2	90.0
Clopidogrel (%)	11.1	10.0
Other antiaggregants (%)	11.1	0.0
***Other***		
Echocardiography (%)	88.9	100
Coronariography (%)	94.4	100
One or two vessel disease (%)	78.6	100
Left Descending Artery disease (%)	50.0	44.4
Left Main Artery disease (%)	6.7	11.1
PTCA (%)	58.8	88.9
CABG (%)	11.1	0

Hx: history of; ACE: angiotensin converting enzyme; PTCA: percutaneous transluminal coronary angioplasty; CABG: coronary artery by-pass grafting.

*p<0.05;

a the proportion of SCAD patients with aspirin treatment was chosen to match the NSTE-ACS group at the moment the sample was taken: overall, 83.3% of ACS patients were given aspirin before blood collection (22.2% were already having aspirin before being taken to hospital).

Almost all patients included in the acute group were high risk patients, 53% having TIMI risk score ≥3, maximal Tn I levels were of 5.1±6.1 ng/mL and maximal CPK-MB of 30.2±39.1 IU/L. Thirty-three per cent of acute patients presented ST and/or T wave changes on the electrocardiogram and in 61% of them these affected to more than four derivations. Almost 44% of patients in the mentioned group presented with segmentary wall motion abnormalities on echocardiography.

### Platelets from NSTE-ACS patients express a characteristic proteomic profile

**Figure 1:**
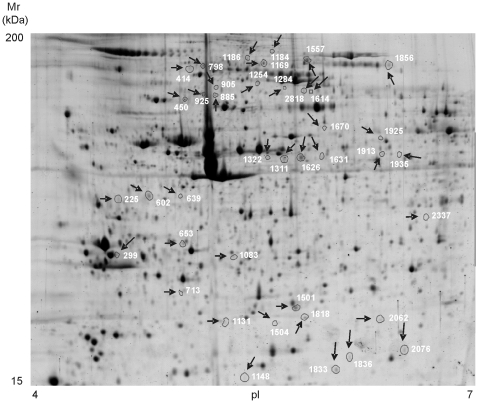
High-resolution 2-DE-based proteome analysis of platelets from NSTE-ACS patients. Representative 2-DE gel image of circulating platelets (IEF: 4–7 pH range; second dimension: 10%SDS-PAGE). The figure shows the location on the 2D gels of those spots differentially regulated when comparing NSTE-ACS patients and SCAD controls. Protein identifications are shown by the identification numbers in Table 2. Further information can be found in Table S1.

As a control of the platelet functional status in each patient, platelet surface expression levels of αIIbβ3 were measured by flow cytometry on PRP. As expected, all subjects displayed normal values of both integrin subunits at the platelet surface, with no significant differences among groups (not shown). Our standardized experimental conditions for platelet isolation and protein extraction resulted in reproducible high-resolution 2D gels (24×21 cm). In consequence, a mean of 2238±112.9 protein features were found on pI 4–7 gels corresponding to platelets from SCAD patients, whereas 2298±88.6 protein features were found in the NSTE-ACS samples. We focused on the identification of disappearing and appearing spots, as well as up- and down-regulation of spot intensities where the fold change was at least 1.5 (with p<0.05). By applying these criteria, 40 protein features were detected as differentially regulated when comparing the platelet proteome of NSTE-ACS patients at admission versus SCAD controls (Figure 1). All protein features were successfully identified by mass spectrometry. They correspond to 22 different open reading frames (ORFs) (Table 2 and Table S1). Ten of the protein features identified were up-regulated in NSTE-ACS samples gels, whereas 30 were down-regulated. Five proteins were represented in the gels by more than one spot; for instance, talin-1 was present in 10 spots. Further information on the 2-DE analysis can be found in Supporting Information S2.

**Table 2:** Platelet proteins differentially regulated in NSTE-ACS versus SCAD patients.

Function	Protein	Uniprot Code	Spot	Fold change
Cytoskeletal	Actin Cytoplasmatic-1	ACTB_HUMAN	925	+1.71
	Alpha-actinin-1	ACTN1_HUMAN	450	+1.63
	Caldesmon	CALD1_HUMAN	2818	+2.25
	F-actin-capping protein subunit beta	CAPZB_HUMAN	1083	−2.04
	Filamin-A	FLNA_HUMAN	1311	−1.62
			1322	−2.86*
			1626	−1.69*
			1631	−2.02*
			1856	+2.16
	Myosin-9	MYH9_HUMAN	885	−1.86
	Talin-1	TLN1_HUMAN	414	−1.97
			639	−2.26
			798	−2.15*
			905	−2.48*
			1169	−1.67*
			1184	+3.36*
			1186	−1.59*
			1254	−2.07*
			1557	−1.70*
			1614	+2.60*
	Tropomyosin alpha chain 3	TPM3_HUMAN	299	−3.90*
	Zyxin	ZYX_HUMAN	602	+1.93*
Signaling	Adenylyl cyclase-associated protein 1	CAP1_HUMAN	2062	−1.73
	FYN-binding protein(ADAP, SLAP-130)	FYB_HUMAN	1148	−1.98
	Integrin-linked protein kinase	ILK_HUMAN	1836	−1.70
			2076	−1.92
	Proto-oncogene tyrosine-protein kinase Src	SRC_HUMAN	1935	−1.57
	Rho GDP-dissociation inhibitor 2	GDIR2_HUMAN	1833	−1.54
Extracellular	Serum Albumin	ALBU_HUMAN	1284	+1.85
			1670	+2.20
			2337	−1.64
	SPARC (Secreted protein acidic and rich in cysteine)	SPRC_HUMAN	225	−2.13*
Vesicles/Secretory trafficking pathway	Dynamin-1-like protein	DNM1L_HUMAN	1925	−1.98*
	Ras-related protein Rab-27B	RB27B_HUMAN	713	−1.66
	Ras-related protein Rab-6B	RAB6B_HUMAN	1131	−1.91*
			1504	−1.54
	Ras-related protein Rab-11A	RB11A_HUMAN	1501	−1.57*
			1818	−1.64
Miscellaneous	MAPRE-1 (Microtubule-associated protein RP/EB family member 1)	MARE1_HUMAN	653	+2.06*
	Septin-11	SEP11_HUMAN	1913	−2.05*

Differentially regulated features have a p<0.05 except those marked with an asterisk, which have a p<0.01. A negative fold change indicates the protein feature is down-regulated in NSTE-ACS whereas a positive fold change indicates the spot is up-regulated in the acute group.

The number of proteins differentially regulated in platelets from NSTE-ACS patients, as compared to stable controls, was decreasing with time. As mentioned above, on admission the number of differentially-regulated protein features was of 40 (Table 2). At day 5, that number was reduced to 28 proteins. After 6 months, only five protein spots remained significantly different (Table S2 and Figure S1).

### Functional groups identified: involvement of proteins related to platelet activation

**Figure 2:**
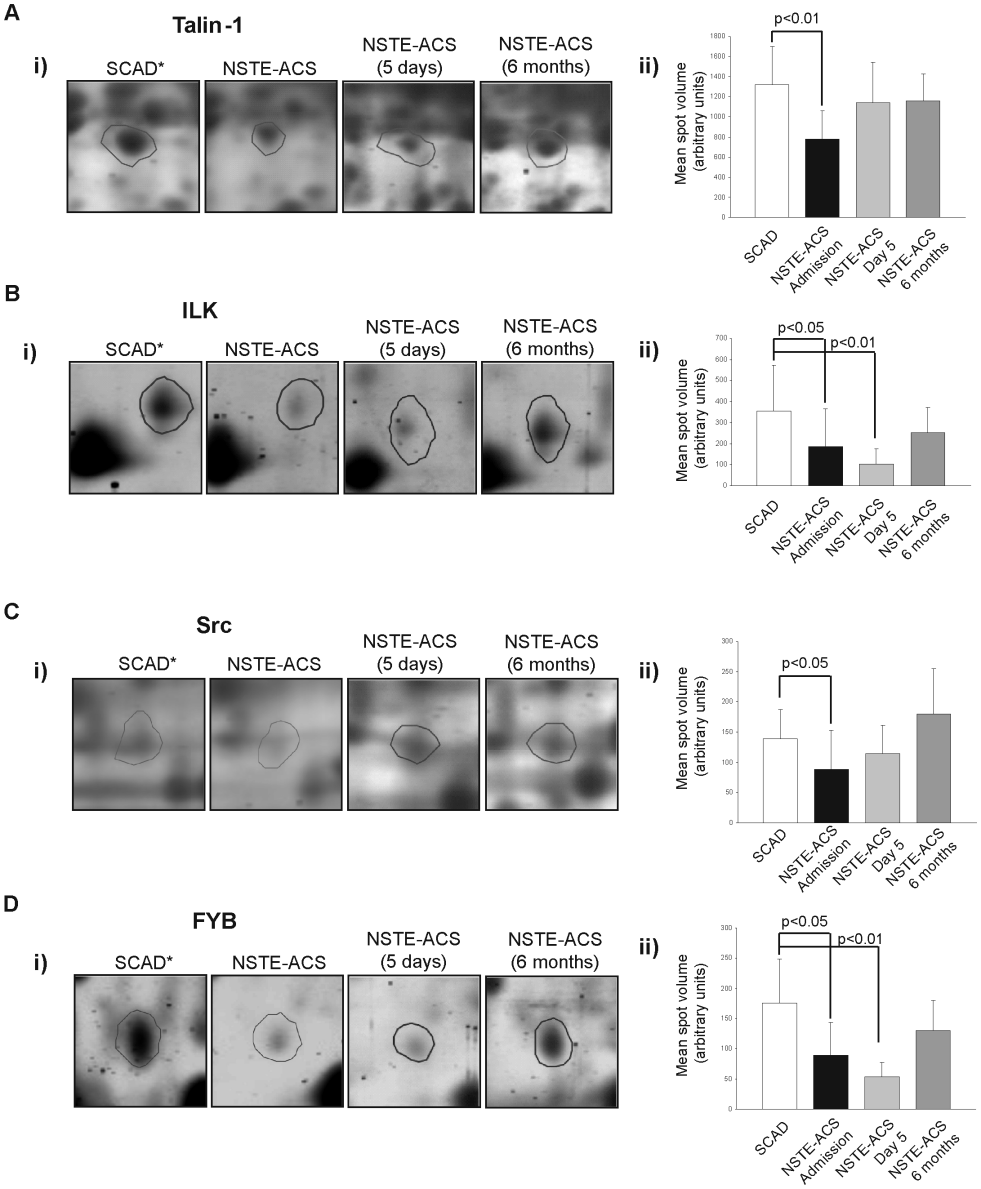
Selection of proteins differentially regulated between NSTE-ACS and SCAD patients. Enlargement of representative spots for (**A**) Talin-1 (spot No. 1557), (**B**) ILK (spot No. 2076), (**C**) Src (spot No. 1935), and (**D**) FYB (spot No. 1148). Panel i) shows representative 2-DE images; panel ii) shows a bar graph for each spot showing variations in mean volume ± SD in different groups: SCAD (n = 10), and NSTE-ACS (n = 18) on admission, day 5, and 6 months after the acute event. * indicates higher expression of the highlighted protein in the referred group of patients.

Attending to their function, the 22 platelet proteins differentially regulated in NSTE-ACS compared to SCAD controls may be classified in the following groups (see Table 2): cytoskeletal/signaling (64%), vesicles/secretory trafficking pathway (18%), extracellular function (9%), and miscellaneous (9%). A close-up view of a selection of differentially regulated proteins, together with their expression levels in controls and NSTE-ACS patients at different time points, is shown in Figure 2.

**Figure 3:**
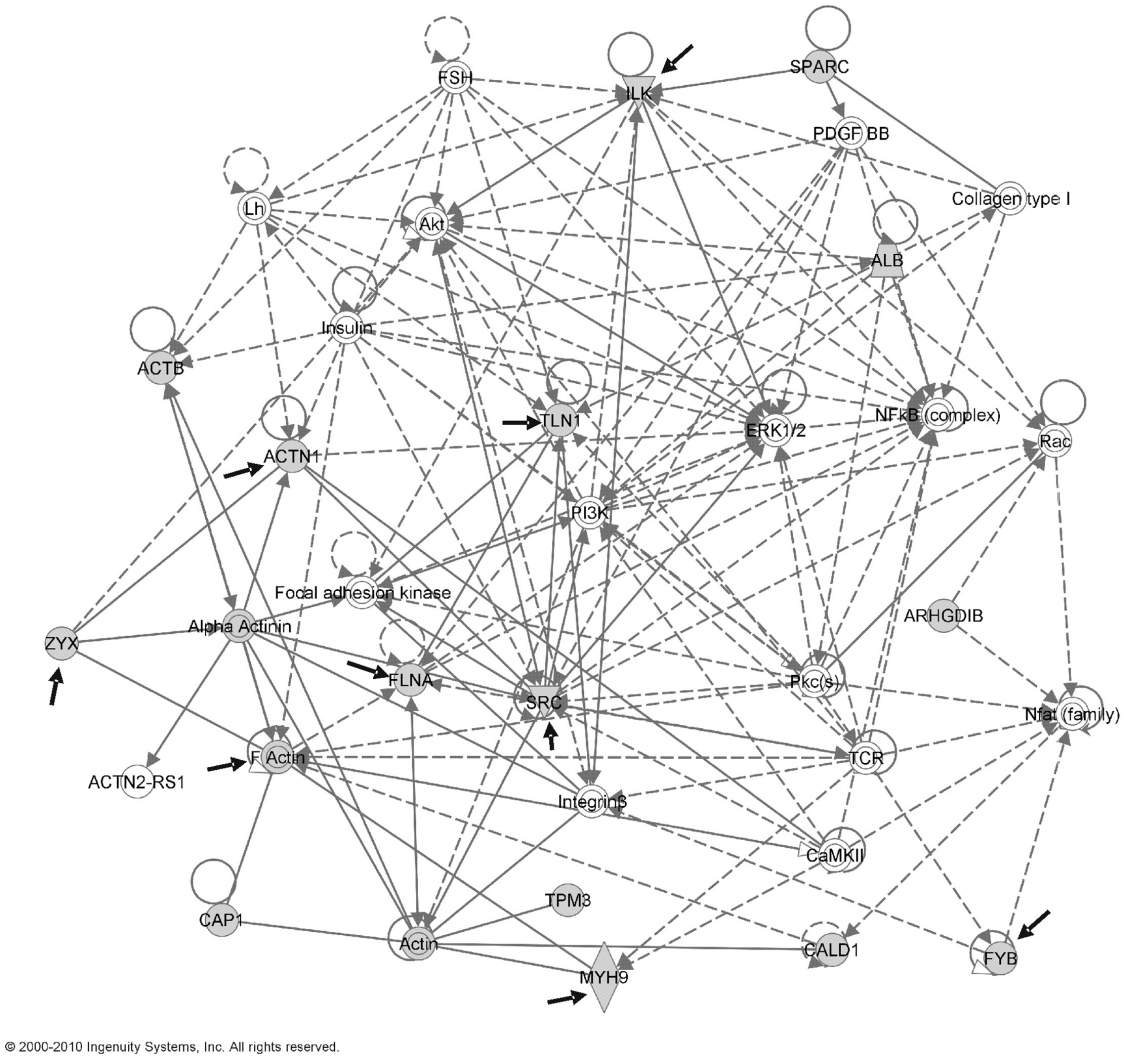
Analysis of differentially regulated proteins by Ingenuity Pathways Analysis software (Ingenuity Systems, CA). Potential protein interactions are shown in the following network: Cellular assembly and organization, cell morphology, and cellular development. Proteins identified by differential analysis are shown as shaded nodes with their gene names. Solid lines represent direct interactions, dotted represent indirect interactions. Arrows from one node to another indicate that this node acts upon the other. Lines without arrows represent binding. Node shapes are: Double circle = complex or group; notched triangle = kinase; wavy shape = enzyme; circle = other. Proteins known to be involved in platelet activation by αIIbβ3 and/or GPVI are indicated with a black solid arrow.

Ingenuity Pathways Analysis software (Ingenuity Systems, CA) was used to investigate possible interactions between all the identified proteins in order to highlight predominant networks. Interestingly, 16 of the 22 differentially regulated proteins identified are interconnected as a part of a common network related to cell assembly and organization and cell morphology (Figure 3). Fourteen of those proteins are either signaling or cytoskeletal, and nine of them are known to play a major role in platelet activation by αIIbβ3 and/or GPVI receptors (Figure 3). More precisely, eight of them play a relevant role in integrin αIIbβ3 signaling: α-actinin-1, ADAP (adhesion- and degranulation promoting adapter protein) – also known as FYB or SLAP-130 - and Src [12]; filamin-A [16], F-actin capping protein [17], zyxin [17], ILK [18], and talin-1 [19]. Interestingly, ILK was also shown to interact with SPARC (*Secreted Protein Acidic and Rich in Cysteine*) (Fig. 3). In addition, five proteins are known to be involved in the GPVI signaling cascade, as demonstrated by a recent phosphoproteomics study of GPVI-activated platelets [11]: α-actinin-1, FYB, ILK, and Src (common to αIIbβ3 signaling), plus myosin-9.

**Figure 4:**
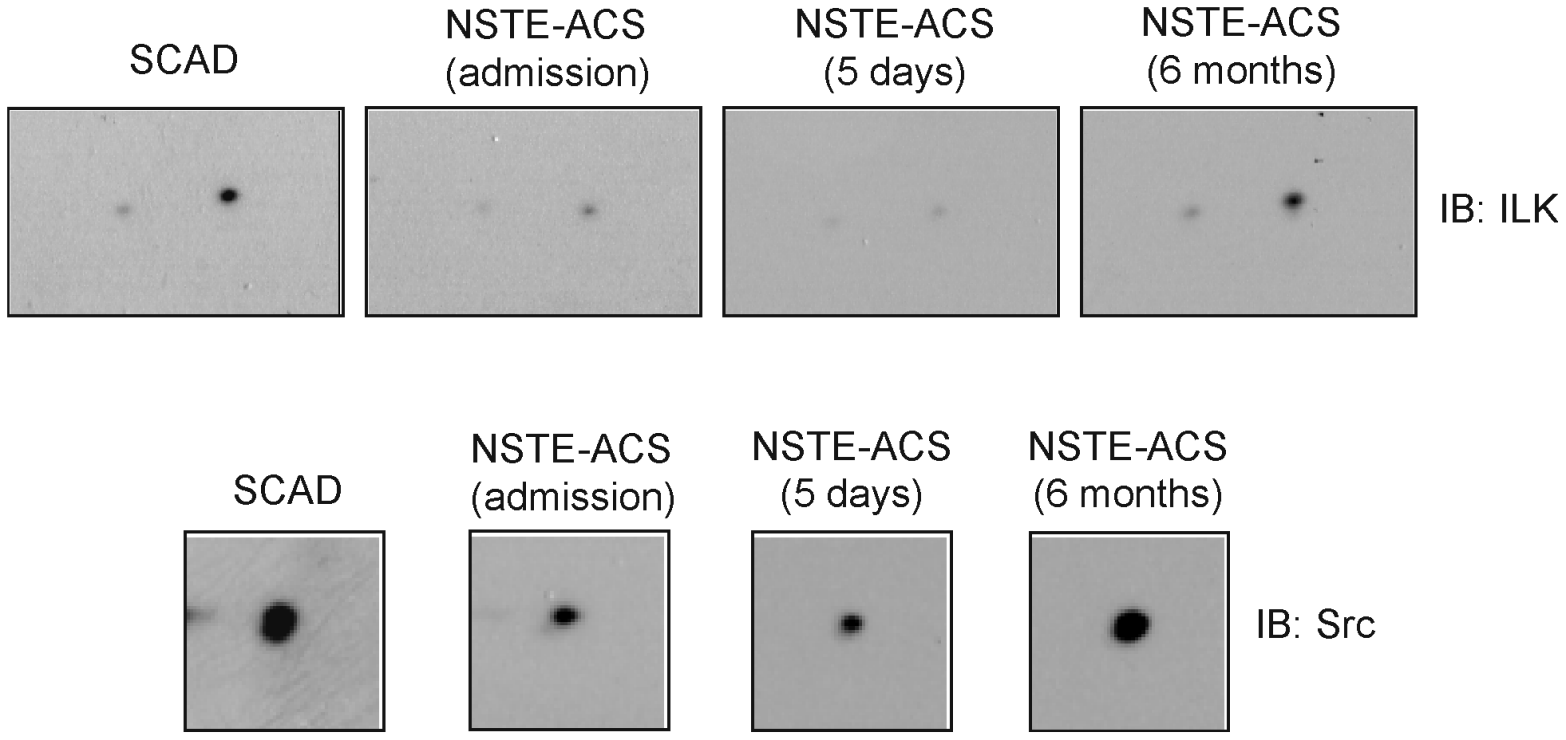
ILK and Src are differentially regulated in NSTE-ACS versus SCAD platelets due to PTMs. Differentially regulated protein features corresponding to ILK and Src are highlighted in 2D-western blot images representative of the results obtained for the patients included in the study. Membranes were probed with mouse antibodies against ILK, and Src.

A selection of the above proteins was validated by 1D and 2D western blotting. We chose two signaling proteins, Src and ILK, and a secreted one, SPARC. No statistically significant differences were observed for Src and ILK by western blot from 1D gels (data not shown). As it can be appreciated by 2D-western blotting, differences related to Src and ILK were on variants originated following post-translational modifications (PTMs), quite common in these signaling proteins after platelet activation: phosphorylation in the case of Src, and proteolysis in the case of ILK (Figure 4). Interestingly, the two ILK spots identified corresponded to N-terminal proteolytic fragments of the protein (Figure 4 and Table S1). A theoretical calculation of pI and molecular weigh on the ExPASy proteomics server (http://www.expasy.org) gave for an ILK N-terminal fragment of 165 amino acids – which is the length comprising the peptides identified by MS – values of 18618.10 Da and pI of 5.95, which are very close to the experimental values obtained in our analysis (Table S1). Those values are obviously far from the theoretical ones for ILK: 51419 Da and pI of 8.30. ILK spots on that range were not detected because the analysis focused on the pI 4–7 range. In the case of Src, the differentially regulated protein feature identified has an experimental pI of 6.0, when the theoretical one is of 7.10 (Table S1), which is consistent with a phosphorylated form.

### Platelet-secreted protein SPARC is down-regulated in platelets from NSTE-ACS patients

**Figure 5:**
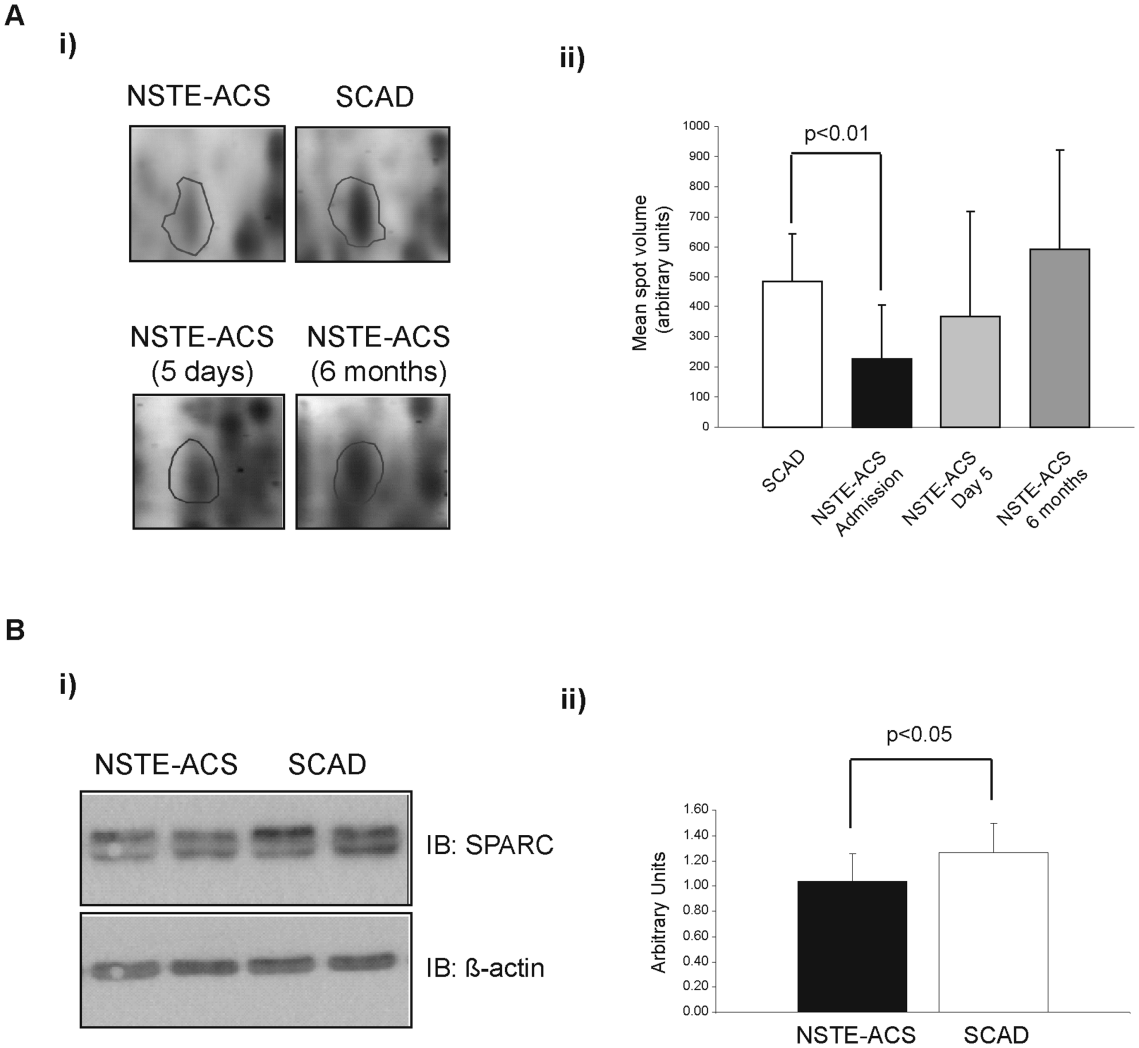
SPARC is down-regulated in platelets from NSTE-ACS patients. **A**) 2-DE proteomics data. Panel i) shows representative 2-DE images for SPARC; panel ii) presents a bar graph showing variations in mean volume ± SD corresponding to SPARC spots in different groups: SCAD (n = 10), and NSTE-ACS (n = 18) on admission, day 5, and 6 months after the acute event. **B**) Western blotting data. i) Western blot analysis of SPARC (upper panel) and β-actin (lower panel) protein expression levels in platelets from NSTE-ACS and SCAD patients. Images are representative of the results obtained for all the patients included in the study. ii) Densitometry graph representing the mean values ± SD of band intensities corresponding to SPARC expression assessed by western blot, with respect to β-actin. Experiments were done in duplicate.

In the proteomic analysis, SPARC was detected in one spot down-regulated in platelets from NSTE-ACS patients. Samples obtained during follow-up revealed that significant differences between acute and stable patients disappeared already at day 5 after the acute event (Figure 5A). Western blot analysis of intracellular SPARC following 1D-SDS PAGE confirmed the proteomics results (Figure 5B). This down-regulation could be due to a secretion process, given the limited protein synthesis capacity of platelets [20]. In this context, preliminary studies on platelet-poor plasma (PPP) from NSTE-ACS patients, compared to matched chronic controls, suggest that circulating SPARC levels are increased in NSTE-ACS patients (not shown).

## Discussion

The present study demonstrates for the first time that platelets from NSTE-ACS patients express a characteristic 2-DE-based proteomic profile during the acute phase compared to platelets from SCAD matched controls, involving a significant number of proteins implicated in platelet signaling. The significant presence of interconnected cytoskeletal, signaling, and secreted proteins among the variants is in agreement with the notion of increased platelet activation in the NSTE-ACS patients in parallel to the acute event. Our proteomic findings regarding the NSTE-ACS patients were confirmed not only by their comparison with a control SCAD group, but also by a prospective re-evaluation in sequential visits during 6-month follow-up.

Platelets are implicated in the initiation, development and total extent of myocardial damage during an acute event [21]. Moreover, there is no doubt platelets play a fundamental role in the thrombus formation that follows the atheroma plaque rupture and leads to an ACS. Indeed, a few platelet-activation markers have been associated with ACS. That is the case of the platelet surface P-selectin or CD40 ligand (CD40-L or CD154) [22]. A recent study has shown patients with acute myocardial infarction (MI) and unstable angina have higher levels of platelet CD154 and P-selectin as compared to those with stable angina or healthy volunteers [23]. Nevertheless, the more direct, biology-based approach to platelet biomarkers may rest with an ability to characterize them in either a state of interaction with other cells (e.g. platelet-leukocyte aggregates) or transformation (e.g. microparticles), or by analyzing protein/mRNA expression (by proteomics and transcriptomics) [22]. In this context, our study tried to pioneer the application of platelet proteomics to the study of ACS with the aim of improving our knowledge on the protein changes that happen in platelets in parallel to the onset of the acute event. We focused our efforts on NSTE-ACS, which is generally associated with white, platelet-rich, and only partially occlusive thrombus, which cause minimal myocardial necrosis [1].

Eighty-five per cent of the platelet proteome is within the 4–7 pI range when separated by 2-DE [24], so we decided to focus on that range. The use of large *zoom* gels and a very sensitive fluorescent dye allowed the detection of over 2,200 proteins per gel. Following our proteomic analysis, we found 40 protein spots to be differentially regulated between platelets from NSTE-ACS and SCAD patients. The fact those spots were related to 22 unique genes suggests some cases of extensive PTMs, quite common in platelets following activation, such as phosphorylations or natural proteolysis. A good example to illustrate the above is talin-1, identified in our study in 10 spots. The NetPhos 2.0 server (Technical University of Denmark, Lyngby, Denmark) predicts a total number of 111 theoretical phosphorylation sites for talin [25]. Phosphorylation of some of those sites and dephosphorylation of others, following platelet activation, could explain the amount of talin features found in the present study with similar molecular weigh and different pI. Regarding the discrepancy between the theoretical molecular mass of talin-1 (269,718 Da) and the in-gel mass, the reason is natural proteolysis by calpain [26].

### Involvement of signaling proteins

It is noteworthy that most of the proteins identified in the present study are interconnected in a common network that primarily involves cytoskeletal and signaling proteins. Platelet activation is associated with specific receptor-ligand interactions that induce outside-in signaling resulting in intracellular phosphorylation, inside-out signaling, cytoskeleton reorganization and granules secretion. This eventually leads to platelet adhesion and aggregation, and thrombus formation. Two of the main platelet receptors are GPVI and the integrin αIIbβ3. GPVI is the main signaling receptor for collagen whereas αIIbβ3 interacts with fibrinogen and is the main responsible for platelet aggregation. Interestingly, an important number of the differentially regulated proteins identified in our study are known to be phosphorylated in response to platelet activation by GPVI and αIIbβ3 [11], [12]. Both receptors share some similarities in the initiation of their signaling cascades, where Src family kinases (SFKs) play a major role. Outside-in signaling by αIIbβ3 is positively regulated by talin, which physically binds to the β3 tail through a phosphotyrosine binding domain (PTB) [19], and Src [12]. Both Src and talin were identified in our study.

In platelets, ILK contributes to inside-out integrin activation [27], and participates in a ternary complex – together with *particularly interesting new Cys-His protein* (PINCH) and parvin – that functions as a signaling platform for integrins [28]. ILK not only controls integrity affinity but is also required for α-granule secretion, and therefore may play a central role in the regulation of platelet function [18]. Interestingly, we describe here for the first time the identification of N-terminal proteolytic fragments of ILK containing the PINCH binding domain. This opens new windows for future functional studies related to platelet activation by receptors, such as the integrin αIIbβ3, where ILK plays a major role.

### Secreted proteins: SPARC

An important event associated with platelet activation is the release of proteins. In our analysis we identified several proteins either secreted or involved in secretory trafficking pathways. In addition, platelet activation is associated with an increase in the release of microparticles (MPs). Indeed, increased numbers of circulating MPs have been reported in patients with ACS [29], and platelet-derived MPs have been shown to induce angiogenesis and improve revascularization after chronic ischemia *in vivo* [30]. Interestingly, 91% of the proteins identified in our study have been reported to be present in platelet MPs [31], [32], and 50% in the platelet releasate [31]. We hypothesize that some of those proteins could be released from the activated platelets either by direct secretion or as a part of MPs. The limited protein synthesis capacity of platelets [20] could explain why some of those proteins (e.g. SPARC) are down-regulated in NSTE-ACS patients.

As mentioned above, one of the secreted proteins identified in our study was SPARC, which is primarily released from α-granules following platelet activation [33] and is also present in platelet MPs [31]. SPARC is a matricellular glycoprotein transiently induced in the infarct, regulating cellular interactions and promoting matrix assembly. It is also involved in wound healing by the facilitation of angiogenesis [34]. Recent studies have shown that local production of SPARC is essential for maintenance of the integrity of cardiac ECM after MI, emphasizing the potential therapeutic application of this protein to prevent cardiac dilatation and dysfunction after MI [35]. Interestingly, SPARC regulates ECM organization through its modulation of ILK activity [36]. On the other hand, in vascular endothelial cells exogenous SPARC facilitates endothelial permeability resulting in barrier dysfunction. Indeed, SPARC has been observed in atherosclerotic regions, and SPARC plasma levels were found to be elevated in obesity and CAD patients [37]. The possibility that SPARC may be ectopically expressed in those patients, such as in the activated platelets, cannot be excluded [37]. Our data suggest a higher platelet secretion of SPARC in NSTE-ACS patients. Further research is on the way with plasma samples from a larger number of patients and controls to confirm this hypothesis. In that case, it would be also of interest to explore whether platelets release SPARC just after or before the acute event, which could be related to its primary physiological role in the disease.

### Study limitations

The study presents some limitations that should be taken in mind. Ideally, NSTE-ACS patients entering the study should have been with no drug therapy that could interfere with the analysis so the control group would have been healthy subjects; however that was not feasible for obvious reasons. In order to compensate for this factor, we chose a control group consisting in SCAD patients matched in the best possible way to the acute group. Secondly, the small volume of blood available per patient did not allow running a higher number of gels and to cover more basic pI ranges in the 2-DE analysis, so some additional differences could have been missed. In addition, there are limitations inherent to 2-DE, such as the potential under-representation of very hydrophobic proteins. Sample limitation was also an impediment to do further functional analyses related to the proteins identified; more samples will be needed to do so. Further characterization of the platelet proteome, focusing on additional subgroups of ACS patients, will likely increase our understanding of platelet functions in ACS.

### Conclusions

This study provides novel information on platelet protein changes associated to NSTE-ACS and shows proteomics can be used to follow platelet proteome changes during the disease follow-up. The proteomic analysis identified several differentially regulated proteins integrated in a common network linked to changes in cell morphology. Several of those proteins are involved in signal transduction; indeed some are known to participate in αIIbβ3 and GPVI signaling, which suggests these pathways may play a relevant role in platelet activation associated with NSTE-ACS. Interestingly, the levels of platelet-secreted glycoprotein SPARC were decreased in platelets from NSTE-ACS patients as compared to stable controls, which is consistent with a platelet secretion associated to platelet activation in NSTE-ACS. Our results, if confirmed in larger studies, open a field for future investigation on novel therapeutic targets in ACS.

## Supporting Information

Figure S1 Follow-up: Platelet protein differences between NSTE-ACS and SCAD patients decreased with time after the acute event.
(0.06 MB PDF)

Table S1 Additional data on MS protein identification by MALDI-MS.
(1.13 MB DOC)

Table S2 List of proteins differentially regulated in NSTE-ACS patients' platelets at different times in comparison with stable patients (SCAD).
(0.07 MB DOC)

Supporting Information S1 (0.03 MB DOC)

Supporting Information S2 (2.99 MB PDF)
