# Correction: Proteins Involved in Platelet Signaling Are Differentially Regulated in Acute Coronary Syndrome: A Proteomic Study

**DOI:** 10.1371/annotation/9ef9bdf1-098e-438b-9c97-62f5dd4deb60

**Published:** 2011-02-25

**Authors:** Andrés Fernández Parguiña, Lilian Grigorian-Shamajian, Rosa M. Agra, Elvis Teijeira-Fernández, Isaac Rosa, Jana Alonso, Juan E. Viñuela-Roldán, Ana Seoane, José Ramón González-Juanatey, Ángel García

The first author's name was incorrectly written in the citation and copyright statement. The correct citation is: Pargui&ntilde;a AF, Grigorian-Shamajian L, Agra RM, Teijeira-Fern&aacute;ndez E, Rosa I, et al. (2010) Proteins Involved in Platelet Signaling Are Differentially Regulated in Acute Coronary Syndrome: A Proteomic Study. PLoS ONE 5(10): e13404. doi:10.1371/journal.pone.0013404 The correct copyright is: Copyright: &copy; 2010 Pargui&ntilde;a et al. This is an open-access article distributed under the terms of the Creative Commons Attribution License, which permits unrestricted use, distribution, and reproduction in any medium, provided the original author and source are credited.

