# Supplementary material for: Proteins Involved in Platelet Signaling Are Differentially Regulated in Acute Coronary Syndrome: A Proteomic Study
Source: PLoS One. 2010 Oct 14;5(10):e13404. doi: 10.1371/journal.pone.0013404 (PMC2954807; doi:10.1371/journal.pone.0013404)
Supplement: Figure S1 — Follow-up: Platelet protein differences between NSTE-ACS and SCAD patients decreased with time after the acute event. (0.06 MB PDF) [file pone.0013404.s001.pdf]

Figure S1. Follow-up.

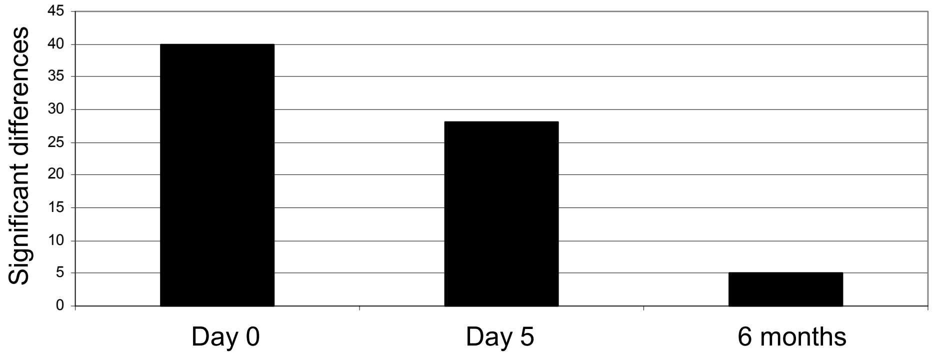

Platelet protein differences between NSTE-ACS and SCAD patients decreased with time after the acute event.
