# Supplementary material for: Proteins Involved in Platelet Signaling Are Differentially Regulated in Acute Coronary Syndrome: A Proteomic Study
Source: PLoS One. 2010 Oct 14;5(10):e13404. doi: 10.1371/journal.pone.0013404 (PMC2954807; doi:10.1371/journal.pone.0013404)
Supplement: Table S1 — Additional data on MS protein identification by MALDI-MS. (1.13 MB DOC) [file pone.0013404.s002.doc]

***Table S1. Additional data on MS protein identification by MALDI-MS.***

| **Spot** | **N^†^ / %^‡^** | **Mascot Score** | **Peptides identified by MS** | | **Identified protein** | **Accession number** | **Mean spot volume NSTE-ACS ± SD / Mean spot volume SCAD ± SD** | **MW (exp) / MW (theo)** | **pI (exp) / pI (theo)** | **Fold change** |
| --- | --- | --- | --- | --- | --- | --- | --- | --- | --- | --- |
|  |  |  | **M+H** | **Sequence** |  |  |  |  |  |  |
| 225 | 7/24 | 285 | 1106.63 | *NVLVTLYER | Secreted protein acidic and rich in cysteine | SPRC_HUMAN | 226.31 ± 178.24 / 482.67 ± 160.25 | 38266 / 32698 | 4.75 / 4.66 | -2.13^§^ |
|  |  |  | 1419.77 | *LEAGDHPVELLAR |  |  |  |  |  |  |
|  |  |  | 1441.70 | APLIPMEHCTTR |  |  |  |  |  |  |
|  |  |  | 1447.65 | TFDSSCHFFATK |  |  |  |  |  |  |
|  |  |  | 1575.87 | *RLEAGDHPVELLAR |  |  |  |  |  |  |
|  |  |  | 1949.97 | YIPPCLDSELTEFPLR |  |  |  |  |  |  |
|  |  |  | 2278.14 | NVLVTLYERDEDNNLLTEK |  |  |  |  |  |  |
| 299 | 14/30 | 421 | 722.34 | AEFAER | Tropomyosin alpha-3 chain | TPM3_HUMAN | 573.84 ± 852.34 / 2240.21 ± 1286.72 | 28029 / 32819 | 4.71 / 4.68 | -3.90^§^ |
|  |  |  | 744.45 | LATALQK |  |  |  |  |  |  |
|  |  |  | 766.37 | YEEVAR |  |  |  |  |  |  |
|  |  |  | 777.34 | AADESER |  |  |  |  |  |  |
|  |  |  | 875.45 | SLEAQAEK |  |  |  |  |  |  |
|  |  |  | 894.47 | YEEVARK |  |  |  |  |  |  |
|  |  |  | 940.45 | HIAEEADR |  |  |  |  |  |  |
|  |  |  | 1147.60 | MELQEIQLK |  |  |  |  |  |  |
|  |  |  | 1156.66 | *LVIIEGDLER |  |  |  |  |  |  |
|  |  |  | 1243.65 | *IQLVEEELDR |  |  |  |  |  |  |
|  |  |  | 1284.75 | *KLVIIEGDLER |  |  |  |  |  |  |
|  |  |  | 1316.64 | *AADAEAEVASLNR |  |  |  |  |  |  |
|  |  |  | 1399.75 | *RIQLVEEELDR |  |  |  |  |  |  |
|  |  |  | 1472.74 | AADAEAEVASLNRR |  |  |  |  |  |  |
| 414 | 14/8 | 309 | 864.50 | KLITSMR | Talin-1 | TLN1_HUMAN | 107.88 ± 65.41 / 212.21 ± 92.11 | 117983 / 269767 | 5.11 / 5.77 | -1.97 |
|  |  |  | 1011.63 | KLEQLKPR |  |  |  |  |  |  |
|  |  |  | 1057.60 | NLGTALAELR |  |  |  |  |  |  |
|  |  |  | 1085.64 | GLAGAVSELLR |  |  |  |  |  |  |
|  |  |  | 1349.62 | AAGHPGDPESQQR |  |  |  |  |  |  |
|  |  |  | 1492.88 | *AVAEQIPLLVQGVR |  |  |  |  |  |  |
|  |  |  | 1523.68 | GAAAHPDSEEQQQR |  |  |  |  |  |  |
|  |  |  | 2027.05 | *LNEAAAGLNQAATELVQASR |  |  |  |  |  |  |
|  |  |  | 2034.01 | LLSDSLPPSTGTFQEAQSR |  |  |  |  |  |  |
|  |  |  | 2061.99 | AVEGCVSASQAATEDGQLLR |  |  |  |  |  |  |
|  |  |  | 2122.18 | LLAALLEDEGGSGRPLLQAAK |  |  |  |  |  |  |
|  |  |  | 2140.11 | VVAPTISSPVCQEQLVEAGR |  |  |  |  |  |  |
|  |  |  | 2573.32 | *AVSSAIAQLLGEVAQGNENYAGIAAR |  |  |  |  |  |  |
|  |  |  | 2733.20 | FGQDFSTFLEAGVEMAGQAPSQEDR |  |  |  |  |  |  |
| 450 | 29/37 | 518 | 864.50 | ALDFIASK | Alpha-actinin-1 | ACTN1_HUMAN | 135.43 ± 63.65 / 82.91 ± 38.86 | 76753 / 103058 | 5.09 / 5.25 | +1.63 |
|  |  |  | 993.46 | ASFNHFDR |  |  |  |  |  |  |
|  |  |  | 1028.55 | TIPWLENR |  |  |  |  |  |  |
|  |  |  | 1044.55 | IMSIVDPNR |  |  |  |  |  |  |
|  |  |  | 1113.53 | LEDFRDYR |  |  |  |  |  |  |
|  |  |  | 1115.56 | ATLPDADKER |  |  |  |  |  |  |
|  |  |  | 1170.55 | NYITMDELR |  |  |  |  |  |  |
|  |  |  | 1215.67 | LASDLLEWIR |  |  |  |  |  |  |
|  |  |  | 1293.75 | LAILGIHNEVSK |  |  |  |  |  |  |
|  |  |  | 1316.64 | DHSGTLGPEEFK |  |  |  |  |  |  |
|  |  |  | 1317.60 | HTNYTMEHIR |  |  |  |  |  |  |
|  |  |  | 1325.66 | RDQALTEEHAR |  |  |  |  |  |  |
|  |  |  | 1354.61 | GISQEQMNEFR |  |  |  |  |  |  |
|  |  |  | 1386.77 | *VGWEQLLTTIAR |  |  |  |  |  |  |
|  |  |  | 1421.71 | GYEEWLLNEIR |  |  |  |  |  |  |
|  |  |  | 1429.77 | *TINEVENQILTR |  |  |  |  |  |  |
|  |  |  | 1477.72 | LSNRPAFMPSEGR |  |  |  |  |  |  |
|  |  |  | 1561.73 | *ELPPDQAEYCIAR |  |  |  |  |  |  |
|  |  |  | 1711.91 | LLETIDQLYLEYAK |  |  |  |  |  |  |
|  |  |  | 1746.89 | LGVVTFQAFIDFMSR |  |  |  |  |  |  |
|  |  |  | 1753.83 | KHEAFESDLAAHQDR |  |  |  |  |  |  |
|  |  |  | 1767.89 | ILAGDKNYITMDELR |  |  |  |  |  |  |
|  |  |  | 1775.85 | MLDAEDIVGTARPDEK |  |  |  |  |  |  |
|  |  |  | 2023.97 | AIMTYVSSFYHAFSGAQK |  |  |  |  |  |  |
|  |  |  | 2067.95 | VLAVNQENEQLMEDYEK |  |  |  |  |  |  |
|  |  |  | 2291.04 | ASFNHFDRDHSGTLGPEEFK |  |  |  |  |  |  |
|  |  |  | 2339.18 | IDQLEGDHQLIQEALIFDNK |  |  |  |  |  |  |
|  |  |  | 2396.12 | *ACLISLGYDIGNDPQGEAEFAR |  |  |  |  |  |  |
|  |  |  | 2721.30 | AIMTYVSSFYHAFSGAQKAETAANR |  |  |  |  |  |  |
| 602 | 7/16 | 221 | 1039.63 | *FGPVVAPKPK | Zyxin | ZYX_HUMAN | 360.19 ± 211.79 / 186.84 ± 108.69 | 37638 / 61146 | 4.96 / 6.23 | +1.93^§^ |
|  |  |  | 1719.90 | *VSSGYVPPPVATPFSSK |  |  |  |  |  |  |
|  |  |  | 1832.95 | GPPASSPAPAPKFSPVTP |  |  |  |  |  |  |
|  |  |  | 1989.00 | *VNPFRPGDSEPPPAPGAQR |  |  |  |  |  |  |
|  |  |  | 2197.08 | VSSIDLEIDSLSSLLDDMTK |  |  |  |  |  |  |
|  |  |  | 2454.23 | EKVSSIDLEIDSLSSLLDDMTK |  |  |  |  |  |  |
|  |  |  | 2798.36 | VSSIDLEIDSLSSLLDDMTKNDPFK |  |  |  |  |  |  |
| 639 | 10/5 | 556 | 864.49 | KLITSMR | Talin-1 | TLN1_HUMAN | 62.64 ± 38.73 / 141.63 ± 116.97 | 39183 / 269767 | 5.07 / 5.77 | -2.26 |
|  |  |  | 965.50 | AHATGAGPAGR |  |  |  |  |  |  |
|  |  |  | 1011.63 | KLEQLKPR |  |  |  |  |  |  |
|  |  |  | 1013.51 | *SAQPASAEPR |  |  |  |  |  |  |
|  |  |  | 1084.66 | AVASAAAALVLK |  |  |  |  |  |  |
|  |  |  | 1085.64 | *GLAGAVSELLR |  |  |  |  |  |  |
|  |  |  | 2061.99 | *AVEGCVSASQAATEDGQLLR |  |  |  |  |  |  |
|  |  |  | 2091.16 | *GVGAAATAVTQALNELLQHVK |  |  |  |  |  |  |
|  |  |  | 2122.19 | *LLAALLEDEGGSGRPLLQAAK |  |  |  |  |  |  |
|  |  |  | 2140.11 | *VVAPTISSPVCQEQLVEAGR |  |  |  |  |  |  |
| 653 | 7/35 | 339 | 1231.65 | *LTVEDLEKER | Microtubule-associated protein RP/EB family member 1 | MARE1_HUMAN | 170.95 ± 70.34 / 83.00 ± 36.62 | 29781 / 29868 | 5.08 / 5.02 | +2.06^§^ |
|  |  |  | 1320.65 | *LEHEYIQNFK |  |  |  |  |  |  |
|  |  |  | 1925.05 | *KPLTSSSAAPQRPISTQR |  |  |  |  |  |  |
|  |  |  | 1963.88 | *FFDANYDGKDYDPVAAR |  |  |  |  |  |  |
|  |  |  | 2019.12 | QGQETAVAPSLVAPALNKPK |  |  |  |  |  |  |
|  |  |  | 2270.08 | *NIELICQENEGENDPVLQR |  |  |  |  |  |  |
|  |  |  | 2655.28 | GAGYTFGQDISETFNHANGLTLVSR |  |  |  |  |  |  |
| 713 | 11/52 | 381 | 800.43 | *TTFLYR | Ras-related protein Rab-27B | RB27B_HUMAN | 134.42 ± 86.34 / 223.64 ± 80.11 | 23776 / 24477 | 5.07 / 5.35 | -1.66 |
|  |  |  | 814.40 | ADLPDQR |  |  |  |  |  |  |
|  |  |  | 942.51 | *SLTTAFFR |  |  |  |  |  |  |
|  |  |  | 1168.64 | *FITTVGIDFR |  |  |  |  |  |  |
|  |  |  | 1377.69 | VVYNAQGPNGSSGK |  |  |  |  |  |  |
|  |  |  | 1417.81 | AVETLLDLIMKR |  |  |  |  |  |  |
|  |  |  | 1533.78 | RVVYNAQGPNGSSGK |  |  |  |  |  |  |
|  |  |  | 1552.79 | *VHLQLWDTAGQER |  |  |  |  |  |  |
|  |  |  | 1974.94 | YGIPYFETSAATGQNVEK |  |  |  |  |  |  |
|  |  |  | 2465.18 | DAMGFLLMFDLTSQQSFLNVR |  |  |  |  |  |  |
|  |  |  | 2531.24 | ELADKYGIPYFETSAATGQNVEK |  |  |  |  |  |  |
| 798 | 34/20 | 845 | 716.40 | SLAQAAR | Talin-1 | TLN1_HUMAN | 443.92 ± 251.21 / 955.30 ± 368.14 | 118657 / 269767 | 5.16 / 5.77 | -2.15^§^ |
|  |  |  | 726.48 | NKLVPR |  |  |  |  |  |  |
|  |  |  | 746.41 | ASDNLVK |  |  |  |  |  |  |
|  |  |  | 787.44 | SQLAAAAR |  |  |  |  |  |  |
|  |  |  | 802.42 | DVDNALR |  |  |  |  |  |  |
|  |  |  | 857.46 | GTPQDLAR |  |  |  |  |  |  |
|  |  |  | 872.51 | AVTQALNR |  |  |  |  |  |  |
|  |  |  | 965.50 | AHATGAGPAGR |  |  |  |  |  |  |
|  |  |  | 1011.62 | KLEQLKPR |  |  |  |  |  |  |
|  |  |  | 1013.52 | SAQPASAEPR |  |  |  |  |  |  |
|  |  |  | 1057.61 | NLGTALAELR |  |  |  |  |  |  |
|  |  |  | 1076.51 | CVSCLPGQR |  |  |  |  |  |  |
|  |  |  | 1085.64 | GLAGAVSELLR |  |  |  |  |  |  |
|  |  |  | 1349.63 | AAGHPGDPESQQR |  |  |  |  |  |  |
|  |  |  | 1455.77 | ASAGPQPLLVQSCK |  |  |  |  |  |  |
|  |  |  | 1477.74 | KAAGHPGDPESQQR |  |  |  |  |  |  |
|  |  |  | 1492.90 | *AVAEQIPLLVQGVR |  |  |  |  |  |  |
|  |  |  | 1523.70 | GAAAHPDSEEQQQR |  |  |  |  |  |  |
|  |  |  | 1620.80 | NGNLPEFGDAISTASK |  |  |  |  |  |  |
|  |  |  | 1995.02 | QAAASATQTIAAAQHAASTPK |  |  |  |  |  |  |
|  |  |  | 2027.06 | *LNEAAAGLNQAATELVQASR |  |  |  |  |  |  |
|  |  |  | 2034.02 | LLSDSLPPSTGTFQEAQSR |  |  |  |  |  |  |
|  |  |  | 2061.99 | *AVEGCVSASQAATEDGQLLR |  |  |  |  |  |  |
|  |  |  | 2091.15 | GVGAAATAVTQALNELLQHVK |  |  |  |  |  |  |
|  |  |  | 2122.19 | *LLAALLEDEGGSGRPLLQAAK |  |  |  |  |  |  |
|  |  |  | 2140.11 | *VVAPTISSPVCQEQLVEAGR |  |  |  |  |  |  |
|  |  |  | 2150.09 | ASVPTIQDQASAMQLSQCAK |  |  |  |  |  |  |
|  |  |  | 2320.15 | AVTDSINQLITMCTQQAPGQK |  |  |  |  |  |  |
|  |  |  | 2405.13 | AQEACGPLEMDSALSVVQNLEK |  |  |  |  |  |  |
|  |  |  | 2469.29 | GVAALTSDPAVQAIVLDTASDVLDK |  |  |  |  |  |  |
|  |  |  | 2573.32 | *AVSSAIAQLLGEVAQGNENYAGIAAR |  |  |  |  |  |  |
|  |  |  | 2733.21 | FGQDFSTFLEAGVEMAGQAPSQEDR |  |  |  |  |  |  |
|  |  |  | 2754.38 | GSQAQPDSPSAQLALIAASQSFLQPGGK |  |  |  |  |  |  |
|  |  |  | 2996.33 | YDQATDTILTVTENIFSSMGDAGEMVR |  |  |  |  |  |  |
| 885 | 15/9 | 604 | 999.57 | GDLPFVVPR | Myosin-9 | MYH9_HUMAN | 118.96 ± 71.52 / 221.66 ± 148.68 | 77916 / 226401 | 5.21 / 5.50 | -1.86 |
|  |  |  | 1155.67 | *RGDLPFVVPR |  |  |  |  |  |  |
|  |  |  | 1215.67 | ASREEILAQAK |  |  |  |  |  |  |
|  |  |  | 1330.74 | LRLEVNLQAMK |  |  |  |  |  |  |
|  |  |  | 1331.61 | QLEEAEEEAQR |  |  |  |  |  |  |
|  |  |  | 1662.80 | ALEEAMEQKAELER |  |  |  |  |  |  |
|  |  |  | 1726.98 | QLLQANPILEAFGNAK |  |  |  |  |  |  |
|  |  |  | 1815.91 | *IAQLEEQLDNETKER |  |  |  |  |  |  |
|  |  |  | 1869.97 | *ANLQIDQINTDLNLER |  |  |  |  |  |  |
|  |  |  | 1950.00 | *LQQELDDLLVDLDHQR |  |  |  |  |  |  |
|  |  |  | 1998.06 | *KANLQIDQINTDLNLER |  |  |  |  |  |  |
|  |  |  | 2080.96 | SMEAEMIQLQEELAAAER |  |  |  |  |  |  |
|  |  |  | 2088.98 | QAQQERDELADEIANSSGK |  |  |  |  |  |  |
|  |  |  | 2207.14 | TRLQQELDDLLVDLDHQR |  |  |  |  |  |  |
|  |  |  | 2472.17 | *IAQLEEELEEEQGNTELINDR |  |  |  |  |  |  |
| 905 | 8/5 | 227 | 1065.53 | TSTPEDFIR | Talin-1 | TLN1_HUMAN | 66.54 ± 55.17 / 65.24 ± 96.56 | 86132 / 269767 | 5.21 / 5.77 | -2.48^§^ |
|  |  |  | 1335.75 | VSHVLAALQAGNR |  |  |  |  |  |  |
|  |  |  | 1463.76 | TMLESAGGLIQTAR |  |  |  |  |  |  |
|  |  |  | 1702.85 | *EGTETFADHREGILK |  |  |  |  |  |  |
|  |  |  | 1782.95 | *DLDQASLAAVSQQLAPR |  |  |  |  |  |  |
|  |  |  | 2203.12 | AVAAGNSCRQEDVIATANLSR |  |  |  |  |  |  |
|  |  |  | 2235.14 | LASEAKPAAVAAENEEIGSHIK |  |  |  |  |  |  |
|  |  |  | 2249.08 | *SNTSPEELGPLANQLTSDYGR |  |  |  |  |  |  |
| 925 | 14/39 | 266 | 795.48 | IIAPPER | Actin cytoplasmatic-1 | ACTB_HUMAN | 136.35 ± 86.12 / 9.64 ± 38.71 | 79397 / 41737 | 5.16 / 5.29 | +1.71 |
|  |  |  | 923.57 | IIAPPERK |  |  |  |  |  |  |
|  |  |  | 976.45 | AGFAGDDAPR |  |  |  |  |  |  |
|  |  |  | 1132.53 | GYSFTTTAER |  |  |  |  |  |  |
|  |  |  | 1198.71 | *AVFPSIVGRPR |  |  |  |  |  |  |
|  |  |  | 1354.62 | DSYVGDEAQSKR |  |  |  |  |  |  |
|  |  |  | 1515.74 | IWHHTFYNELR |  |  |  |  |  |  |
|  |  |  | 1516.71 | QEYDESGPSIVHR |  |  |  |  |  |  |
|  |  |  | 1629.81 | GYSFTTTAEREIVR |  |  |  |  |  |  |
|  |  |  | 1639.84 | LDLAGRDLTDYLMK |  |  |  |  |  |  |
|  |  |  | 1644.81 | QEYDESGPSIVHRK |  |  |  |  |  |  |
|  |  |  | 1790.90 | *SYELPDGQVITIGNER |  |  |  |  |  |  |
|  |  |  | 1954.06 | VAPEEHPVLLTEAPLNPK |  |  |  |  |  |  |
|  |  |  | 2231.08 | DLYANTVLSGGTTMYPGIADR |  |  |  |  |  |  |
| 1083 | 7/23 | 205 | 786.45 | DIVNGLR | F-actin-capping protein subunit beta | CAPZB_HUMAN | 132.88 ± 117.19 / 270.96 ± 140.08 | 27981 / 31219 | 5.26 / 5.36 | -2.04 |
|  |  |  | 1015.59 | *TKDIVNGLR |  |  |  |  |  |  |
|  |  |  | 1108.65 | RLPPQQIEK |  |  |  |  |  |  |
|  |  |  | 1171.60 | *STLNEIYFGK |  |  |  |  |  |  |
|  |  |  | 1262.65 | LVEDMENKIR |  |  |  |  |  |  |
|  |  |  | 1353.64 | SGSGTMNLGGSLTR |  |  |  |  |  |  |
|  |  |  | 1696.84 | KLEVEANNAFDQYR |  |  |  |  |  |  |
| 1131 | 8/49 | 130 | 943.42 | TMYLEDR | Ras-related protein Rab-6B | RAB6B_HUMAN | 55.79 ± 30.44 / 106.62 ± 51.67 | 20699 / 23462 | 5.21 / 5.41 | -1.91^§^ |
|  |  |  | 948.55 | *SLIPSYIR |  |  |  |  |  |  |
|  |  |  | 1176.66 | LVFLGEQSVGK |  |  |  |  |  |  |
|  |  |  | 1202.60 | *QITIEEGEQR |  |  |  |  |  |  |
|  |  |  | 1316.66 | *LQLWDTAGQER |  |  |  |  |  |  |
|  |  |  | 1370.69 | ELSVMFIETSAK |  |  |  |  |  |  |
|  |  |  | 2429.21 | DSTVAVVVYDITNLNSFQQTSK |  |  |  |  |  |  |
|  |  |  | 2492.15 | FMYDSFDNTYQATIGIDFLSK |  |  |  |  |  |  |
| 1148 | 7/7 | 99 | 702.44 | LTGVKGK | FYN-binding protein | FYB_HUMAN | 88.79 ± 54.53 / 175.45 ± 72.79 | 16458 / 85421 | 5.30 / 6.11 | -1.98 |
|  |  |  | 759.46 | LELEKK |  |  |  |  |  |  |
|  |  |  | 770.42 | *YGYVLR |  |  |  |  |  |  |
|  |  |  | 823.41 | EEKDFR |  |  |  |  |  |  |
|  |  |  | 1027.52 | *FKYDGEIR |  |  |  |  |  |  |
|  |  |  | 1327.67 | NEEGKYGYVLR |  |  |  |  |  |  |
|  |  |  | 2216.13 | DLQVKPGESLEVIQTTDDTK |  |  |  |  |  |  |
| 1169 | 30/14 | 530 | 716.39 | ALHYGR | Talin-1 | TLN1_HUMAN | 442.50 ± 182.14 / 739.65 ± 228.29 | 123469 / 269767 | 5.38 / 5.77 | -1.67^§^ |
|  |  |  | 736.42 | LITSMR |  |  |  |  |  |  |
|  |  |  | 740.46 | ALAVNPR |  |  |  |  |  |  |
|  |  |  | 861.48 | FLPSELR |  |  |  |  |  |  |
|  |  |  | 864.49 | KLITSMR |  |  |  |  |  |  |
|  |  |  | 1011.64 | KLEQLKPR |  |  |  |  |  |  |
|  |  |  | 1140.60 | ALEATTEHIR |  |  |  |  |  |  |
|  |  |  | 1162.52 | EGTETFADHR |  |  |  |  |  |  |
|  |  |  | 1242.62 | FLPSELRDEH |  |  |  |  |  |  |
|  |  |  | 1316.69 | QEDVIATANLSR |  |  |  |  |  |  |
|  |  |  | 1335.75 | *VSHVLAALQAGNR |  |  |  |  |  |  |
|  |  |  | 1408.70 | DPPSWSVLAGHSR |  |  |  |  |  |  |
|  |  |  | 1416.78 | LAQAAQSSVATITR |  |  |  |  |  |  |
|  |  |  | 1453.71 | *EAAYHPEVAPDVR |  |  |  |  |  |  |
|  |  |  | 1463.76 | TMLESAGGLIQTAR |  |  |  |  |  |  |
|  |  |  | 1521.84 | TLAESALQLLYTAK |  |  |  |  |  |  |
|  |  |  | 1572.77 | QELAVFCSPEPPAK |  |  |  |  |  |  |
|  |  |  | 1633.84 | VAGSVTELIQAAEAMK |  |  |  |  |  |  |
|  |  |  | 1663.83 | AAAFEEQENETVVVK |  |  |  |  |  |  |
|  |  |  | 1726.88 | TLSHPQQMALLDQTK |  |  |  |  |  |  |
|  |  |  | 1782.94 | *DLDQASLAAVSQQLAPR |  |  |  |  |  |  |
|  |  |  | 1861.95 | MVGGIAQIIAAQEEMLR |  |  |  |  |  |  |
|  |  |  | 1935.93 | EADESLNFEEQILEAAK |  |  |  |  |  |  |
|  |  |  | 1990.05 | MVGGIAQIIAAQEEMLRK |  |  |  |  |  |  |
|  |  |  | 2074.01 | APGQLECETAIAALNSCLR |  |  |  |  |  |  |
|  |  |  | 2195.18 | LGAASLGAEDPETQVVLINAVK |  |  |  |  |  |  |
|  |  |  | 2249.07 | *SNTSPEELGPLANQLTSDYGR |  |  |  |  |  |  |
|  |  |  | 2310.17 | VGAIPANALDDGQWSQGLISAAR |  |  |  |  |  |  |
|  |  |  | 2317.13 | DKAPGQLECETAIAALNSCLR |  |  |  |  |  |  |
|  |  |  | 3386.74 | EGISQEALHTQMLTAVQEISHLIEPLANAAR |  |  |  |  |  |  |
| 1184 | 27/15 | 367 | 716.38 | ALHYGR | Talin-1 | TLN1_HUMAN | 63.90 ± 32.62 / 19.03 ± 20.30 | 143648 / 269767 | 5.41 / 5.77 | +3.36^§^ |
|  |  |  | 720.41 | LITSMR |  |  |  |  |  |  |
|  |  |  | 736.40 | LITSMR |  |  |  |  |  |  |
|  |  |  | 746.40 | ASDNLVK |  |  |  |  |  |  |
|  |  |  | 805.43 | AIADMLR |  |  |  |  |  |  |
|  |  |  | 1011.63 | KLEQLKPR |  |  |  |  |  |  |
|  |  |  | 1065.53 | TSTPEDFIR |  |  |  |  |  |  |
|  |  |  | 1222.58 | ALDGAFTEENR |  |  |  |  |  |  |
|  |  |  | 1242.62 | FLPSELRDEH |  |  |  |  |  |  |
|  |  |  | 1335.75 | VSHVLAALQAGNR |  |  |  |  |  |  |
|  |  |  | 1416.78 | LAQAAQSSVATITR |  |  |  |  |  |  |
|  |  |  | 1453.71 | EAAYHPEVAPDVR |  |  |  |  |  |  |
|  |  |  | 1463.76 | TMLESAGGLIQTAR |  |  |  |  |  |  |
|  |  |  | 1492.89 | AVAEQIPLLVQGVR |  |  |  |  |  |  |
|  |  |  | 1521.86 | TLAESALQLLYTAK |  |  |  |  |  |  |
|  |  |  | 1620.80 | NGNLPEFGDAISTASK |  |  |  |  |  |  |
|  |  |  | 1663.84 | AAAFEEQENETVVVK |  |  |  |  |  |  |
|  |  |  | 1726.88 | TLSHPQQMALLDQTK |  |  |  |  |  |  |
|  |  |  | 1782.94 | *DLDQASLAAVSQQLAPR |  |  |  |  |  |  |
|  |  |  | 1935.92 | EADESLNFEEQILEAAK |  |  |  |  |  |  |
|  |  |  | 1990.05 | MVGGIAQIIAAQEEMLRK |  |  |  |  |  |  |
|  |  |  | 2027.05 | LNEAAAGLNQAATELVQASR |  |  |  |  |  |  |
|  |  |  | 2195.19 | LGAASLGAEDPETQVVLINAVK |  |  |  |  |  |  |
|  |  |  | 2235.15 | LASEAKPAAVAAENEEIGSHIK |  |  |  |  |  |  |
|  |  |  | 2249.07 | *SNTSPEELGPLANQLTSDYGR |  |  |  |  |  |  |
|  |  |  | 2573.28 | AVSSAIAQLLGEVAQGNENYAGIAAR |  |  |  |  |  |  |
|  |  |  | 3470.72 | AATAPLLEAVDNLSAFASNPEFSSIPAQISPEGR |  |  |  |  |  |  |
| 1186 | 18/11 | 299 | 759.45 | AKSVAQR | Talin-1 | TLN1_HUMAN | 146.93 ± 78.01 / 233.25 ± 62.30 | 132990 / 269767 | 5.33 / 5.77 | -1.59^§^ |
|  |  |  | 864.50 | KLITSMR |  |  |  |  |  |  |
|  |  |  | 1011.63 | KLEQLKPR |  |  |  |  |  |  |
|  |  |  | 1057.60 | NLGTALAELR |  |  |  |  |  |  |
|  |  |  | 1085.64 | GLAGAVSELLR |  |  |  |  |  |  |
|  |  |  | 1222.57 | ALDGAFTEENR |  |  |  |  |  |  |
|  |  |  | 1349.64 | AAGHPGDPESQQR |  |  |  |  |  |  |
|  |  |  | 1492.89 | *AVAEQIPLLVQGVR |  |  |  |  |  |  |
|  |  |  | 1523.69 | GAAAHPDSEEQQQR |  |  |  |  |  |  |
|  |  |  | 2027.06 | *LNEAAAGLNQAATELVQASR |  |  |  |  |  |  |
|  |  |  | 2034.01 | LLSDSLPPSTGTFQEAQSR |  |  |  |  |  |  |
|  |  |  | 2062.02 | AAQKAAAFEEQENETVVVK |  |  |  |  |  |  |
|  |  |  | 2091.14 | GVGAAATAVTQALNELLQHVK |  |  |  |  |  |  |
|  |  |  | 2122.19 | *LLAALLEDEGGSGRPLLQAAK |  |  |  |  |  |  |
|  |  |  | 2140.11 | *VVAPTISSPVCQEQLVEAGR |  |  |  |  |  |  |
|  |  |  | 2573.33 | AVSSAIAQLLGEVAQGNENYAGIAAR |  |  |  |  |  |  |
|  |  |  | 2678.26 | ISIGNVVKTMQFEPSTMVYDACR |  |  |  |  |  |  |
|  |  |  | 2733.23 | FGQDFSTFLEAGVEMAGQAPSQEDR |  |  |  |  |  |  |
| 1254 | 23/12 | 261 | 716.39 | ALHYGR | Talin-1 | TLN1_HUMAN | 112.59 ± 113.79 / 233.42 ± 120.95 | 92858 / 269767 | 5.37 / 5.77 | -2.07^§^ |
|  |  |  | 740.46 | ALAVNPR |  |  |  |  |  |  |
|  |  |  | 864.50 | KLITSMR |  |  |  |  |  |  |
|  |  |  | 1011.63 | KLEQLKPR |  |  |  |  |  |  |
|  |  |  | 1065.53 | TSTPEDFIR |  |  |  |  |  |  |
|  |  |  | 1140.61 | ALEATTEHIR |  |  |  |  |  |  |
|  |  |  | 1162.51 | EGTETFADHR |  |  |  |  |  |  |
|  |  |  | 1222.58 | ALDGAFTEENR |  |  |  |  |  |  |
|  |  |  | 1316.68 | QEDVIATANLSR |  |  |  |  |  |  |
|  |  |  | 1335.75 | VSHVLAALQAGNR |  |  |  |  |  |  |
|  |  |  | 1408.70 | DPPSWSVLAGHSR |  |  |  |  |  |  |
|  |  |  | 1416.78 | LAQAAQSSVATITR |  |  |  |  |  |  |
|  |  |  | 1453.71 | EAAYHPEVAPDVR |  |  |  |  |  |  |
|  |  |  | 1463.76 | TMLESAGGLIQTAR |  |  |  |  |  |  |
|  |  |  | 1521.85 | TLAESALQLLYTAK |  |  |  |  |  |  |
|  |  |  | 1633.85 | VAGSVTELIQAAEAMK |  |  |  |  |  |  |
|  |  |  | 1635.85 | AQVVSNLKGISMSSSK |  |  |  |  |  |  |
|  |  |  | 1726.89 | TLSHPQQMALLDQTK |  |  |  |  |  |  |
|  |  |  | 1782.94 | *DLDQASLAAVSQQLAPR |  |  |  |  |  |  |
|  |  |  | 2195.18 | LGAASLGAEDPETQVVLINAVK |  |  |  |  |  |  |
|  |  |  | 2249.08 | *SNTSPEELGPLANQLTSDYGR |  |  |  |  |  |  |
|  |  |  | 2317.13 | DKAPGQLECETAIAALNSCLR |  |  |  |  |  |  |
|  |  |  | 3470.74 | AATAPLLEAVDNLSAFASNPEFSSIPAQISPEGR |  |  |  |  |  |  |
| 1284 | 2/3 | 111 | 927.50 | *YLYEIAR | Serum albumin | ALBU_HUMAN | 36.77 ± 17.70 / 19.85 ± 11.71 | 85320 / 66472 | 5.48 / 5.67 | +1.85 |
|  |  |  | 1639.94 | *KVPQVSTPTLVEVSR |  |  |  |  |  |  |
| 1311 | 12/5 | 101 | 752.39 | FTVETR | Filamin-A | FLNA_HUMAN | 662.53 ± 547.89 / 1072.33 ± 290.65 | 46524 / 280608 | 5.47 / 5.70 | -1.62 |
|  |  |  | 756.41 | AGGPGLER |  |  |  |  |  |  |
|  |  |  | 952.50 | AEFTVETR |  |  |  |  |  |  |
|  |  |  | 1099.58 | GTVEPQLEAR |  |  |  |  |  |  |
|  |  |  | 1301.67 | FNGTHIPGSPFK |  |  |  |  |  |  |
|  |  |  | 1379.68 | YGGPYHIGGSPFK |  |  |  |  |  |  |
|  |  |  | 1417.79 | RLTVSSLQESGLK |  |  |  |  |  |  |
|  |  |  | 1426.76 | EAGAGGLAIAVEGPSK |  |  |  |  |  |  |
|  |  |  | 1502.75 | FVPAEMGTHTVSVK |  |  |  |  |  |  |
|  |  |  | 1533.77 | AEAGVPAEFSIWTR |  |  |  |  |  |  |
|  |  |  | 1756.92 | VTYTPMAPGSYLISIK |  |  |  |  |  |  |
|  |  |  | 2467.19 | *FNEEHIPDSPFVVPVASPSGDAR |  |  |  |  |  |  |
| 1322 | 17/9 | 356 | 756.41 | AGGPGLER | Filamin-A | FLNA_HUMAN | 123.84 ± 86.16 / 353.92 ± 233.11 | 46616 / 280608 | 5.39 / 5.70 | -2.86^§^ |
|  |  |  | 966.46 | AEISFEDR |  |  |  |  |  |  |
|  |  |  | 1094.56 | AEISFEDRK |  |  |  |  |  |  |
|  |  |  | 1301.67 | FNGTHIPGSPFK |  |  |  |  |  |  |
|  |  |  | 1379.68 | *YGGPYHIGGSPFK |  |  |  |  |  |  |
|  |  |  | 1417.80 | RLTVSSLQESGLK |  |  |  |  |  |  |
|  |  |  | 1434.77 | AGNNMLLVGVHGPR |  |  |  |  |  |  |
|  |  |  | 1502.80 | VNQPASFAVSLNGAK |  |  |  |  |  |  |
|  |  |  | 1518.78 | FVPAEMGTHTVSVK |  |  |  |  |  |  |
|  |  |  | 1533.77 | *AEAGVPAEFSIWTR |  |  |  |  |  |  |
|  |  |  | 1756.92 | VTYTPMAPGSYLISIK |  |  |  |  |  |  |
|  |  |  | 2018.03 | IPEISIQDMTAQVTSPSGK |  |  |  |  |  |  |
|  |  |  | 2057.94 | *THEAEIVEGENHTYCIR |  |  |  |  |  |  |
|  |  |  | 2200.12 | LVSNHSLHETSSVFVDSLTK |  |  |  |  |  |  |
|  |  |  | 2296.16 | GAGSYTIMVLFADQATPTSPIR |  |  |  |  |  |  |
|  |  |  | 2304.16 | GQHVPGSPFQFTVGPLGEGGAHK |  |  |  |  |  |  |
|  |  |  | 2467.19 | *FNEEHIPDSPFVVPVASPSGDAR |  |  |  |  |  |  |
| 1501 | 9/40 | 281 | 858.44 | AVPTDEAR | Ras-related protein Rab-11A | RB11A_HUMAN | 491.19 ± 225.84 / 769.66 ± 179.24 | 21592 / 24393 | 5.62 / 6.12 | -1.57^§^ |
|  |  |  | 944.49 | *AITSAYYR |  |  |  |  |  |  |
|  |  |  | 1080.57 | *STIGVEFATR |  |  |  |  |  |  |
|  |  |  | 1160.57 | *HLTYENVER |  |  |  |  |  |  |
|  |  |  | 1191.59 | IVSQKQMSDR |  |  |  |  |  |  |
|  |  |  | 1274.62 | *AQIWDTAGQER |  |  |  |  |  |  |
|  |  |  | 1289.75 | GAVGALLVYDIAK |  |  |  |  |  |  |
|  |  |  | 1641.83 | DHADSNIVIMLVGNK |  |  |  |  |  |  |
|  |  |  | 2040.05 | ELRDHADSNIVIMLVGNK |  |  |  |  |  |  |
| 1504 | 6/25 | 76 | 719.45 | QLFRR | Ras-related protein Rab-6B | RAB6B_HUMAN | 47.12 ± 29.01 / 72.58 ± 35.13 | 20309 / 23462 | 5.41 / 5.41 | -1.54 |
|  |  |  | 818.44 | TDLADKR |  |  |  |  |  |  |
|  |  |  | 948.55 | *SLIPSYIR |  |  |  |  |  |  |
|  |  |  | 1176.66 | LVFLGEQSVGK |  |  |  |  |  |  |
|  |  |  | 1202.60 | QITIEEGEQR |  |  |  |  |  |  |
|  |  |  | 1316.66 | LQLWDTAGQER |  |  |  |  |  |  |
| 1557 | 18/10 | 386 | 918.50 | EEITGTLR | Talin-1 | TLN1_HUMAN | 777.82 ± 282.93 / 1318.44 ± 382.71 | 130734 / 269767 | 5.62 / 5.77 | -1.70^§^ |
|  |  |  | 1222.57 | ALDGAFTEENR |  |  |  |  |  |  |
|  |  |  | 1242.61 | FLPSELRDEH |  |  |  |  |  |  |
|  |  |  | 1316.67 | QEDVIATANLSR |  |  |  |  |  |  |
|  |  |  | 1335.74 | VSHVLAALQAGNR |  |  |  |  |  |  |
|  |  |  | 1416.76 | LAQAAQSSVATITR |  |  |  |  |  |  |
|  |  |  | 1430.73 | DVDNALRAVGDASK |  |  |  |  |  |  |
|  |  |  | 1453.71 | EAAYHPEVAPDVR |  |  |  |  |  |  |
|  |  |  | 1463.74 | TMLESAGGLIQTAR |  |  |  |  |  |  |
|  |  |  | 1782.94 | *DLDQASLAAVSQQLAPR |  |  |  |  |  |  |
|  |  |  | 1861.96 | MVGGIAQIIAAQEEMLR |  |  |  |  |  |  |
|  |  |  | 1935.93 | *EADESLNFEEQILEAAK |  |  |  |  |  |  |
|  |  |  | 1990.04 | MVGGIAQIIAAQEEMLRK |  |  |  |  |  |  |
|  |  |  | 2195.20 | LGAASLGAEDPETQVVLINAVK |  |  |  |  |  |  |
|  |  |  | 2235.17 | LASEAKPAAVAAENEEIGSHIK |  |  |  |  |  |  |
|  |  |  | 2249.08 | *SNTSPEELGPLANQLTSDYGR |  |  |  |  |  |  |
|  |  |  | 2292.19 | IGITNHDEYSLVRELMEEK |  |  |  |  |  |  |
|  |  |  | 2363.15 | LKPLPGETMEKCTQDLGNSTK |  |  |  |  |  |  |
| 1614 | 15/8 | 292 | 726.47 | NKLVPR | Talin-1 | TLN1_HUMAN | 113.35 ± 79.82 / 43.67 ± 15.29 | 82829 / 269767 | 5.64 / 5.77 | +2.60^§^ |
|  |  |  | 728.47 | KLAQIR |  |  |  |  |  |  |
|  |  |  | 745.45 | DKTLLR |  |  |  |  |  |  |
|  |  |  | 1013.51 | SAQPASAEPR |  |  |  |  |  |  |
|  |  |  | 1031.51 | ERELEEAR |  |  |  |  |  |  |
|  |  |  | 1057.60 | NLGTALAELR |  |  |  |  |  |  |
|  |  |  | 1085.63 | GLAGAVSELLR |  |  |  |  |  |  |
|  |  |  | 1395.74 | VEHGSVALPAIMR |  |  |  |  |  |  |
|  |  |  | 1492.89 | *AVAEQIPLLVQGVR |  |  |  |  |  |  |
|  |  |  | 1523.69 | GAAAHPDSEEQQQR |  |  |  |  |  |  |
|  |  |  | 2061.99 | *AVEGCVSASQAATEDGQLLR |  |  |  |  |  |  |
|  |  |  | 2122.18 | *LLAALLEDEGGSGRPLLQAAK |  |  |  |  |  |  |
|  |  |  | 2140.10 | *VVAPTISSPVCQEQLVEAGR |  |  |  |  |  |  |
|  |  |  | 2235.12 | LASEAKPAAVAAENEEIGSHIK |  |  |  |  |  |  |
|  |  |  | 2573.33 | *AVSSAIAQLLGEVAQGNENYAGIAAR |  |  |  |  |  |  |
| 1626 | 23/11 | 620 | 756.40 | AGGPGLER | Filamin-A | FLNA_HUMAN | 1481.82 ± 920.41 / 2505.48 ± 616.78 | 46616 / 280608 | 5.56 / 5.70 | -1.69^§^ |
|  |  |  | 966.46 | AEISFEDR |  |  |  |  |  |  |
|  |  |  | 1094.55 | AEISFEDRK |  |  |  |  |  |  |
|  |  |  | 1149.62 | ENGVYLIDVK |  |  |  |  |  |  |
|  |  |  | 1261.70 | LTVSSLQESGLK |  |  |  |  |  |  |
|  |  |  | 1301.66 | FNGTHIPGSPFK |  |  |  |  |  |  |
|  |  |  | 1379.67 | *YGGPYHIGGSPFK |  |  |  |  |  |  |
|  |  |  | 1417.80 | RLTVSSLQESGLK |  |  |  |  |  |  |
|  |  |  | 1426.75 | EAGAGGLAIAVEGPSK |  |  |  |  |  |  |
|  |  |  | 1460.52 | MDCQECPEGYR |  |  |  |  |  |  |
|  |  |  | 1502.79 | VNQPASFAVSLNGAK |  |  |  |  |  |  |
|  |  |  | 1518.76 | FVPAEMGTHTVSVK |  |  |  |  |  |  |
|  |  |  | 1533.77 | *AEAGVPAEFSIWTR |  |  |  |  |  |  |
|  |  |  | 1687.68 | VKMDCQECPEGYR |  |  |  |  |  |  |
|  |  |  | 1756.92 | *VTYTPMAPGSYLISIK |  |  |  |  |  |  |
|  |  |  | 1778.90 | GTVEPQLEARGDSTYR |  |  |  |  |  |  |
|  |  |  | 2018.01 | IPEISIQDMTAQVTSPSGK |  |  |  |  |  |  |
|  |  |  | 2057.94 | *THEAEIVEGENHTYCIR |  |  |  |  |  |  |
|  |  |  | 2200.13 | LVSNHSLHETSSVFVDSLTK |  |  |  |  |  |  |
|  |  |  | 2222.12 | APLRVQVQDNEGCPVEALVK |  |  |  |  |  |  |
|  |  |  | 2304.16 | *GQHVPGSPFQFTVGPLGEGGAHK |  |  |  |  |  |  |
|  |  |  | 2467.19 | *FNEEHIPDSPFVVPVASPSGDAR |  |  |  |  |  |  |
|  |  |  | 2766.31 | VHSPSGALEECYVTEIDQDKYAVR |  |  |  |  |  |  |
| 1631 | 14/7 | 294 | 728.46 | VTVKGPK | Filamin-A | FLNA_HUMAN | 210.45 ± 103.02 / 424.37 ± 173.69 | 46801 / 280608 | 5.68 / 5.70 | -2.02^§^ |
|  |  |  | 756.40 | AGGPGLER |  |  |  |  |  |  |
|  |  |  | 761.46 | LVSIDSK |  |  |  |  |  |  |
|  |  |  | 966.46 | AEISFEDR |  |  |  |  |  |  |
|  |  |  | 1301.67 | FNGTHIPGSPFK |  |  |  |  |  |  |
|  |  |  | 1379.68 | *YGGPYHIGGSPFK |  |  |  |  |  |  |
|  |  |  | 1502.80 | VNQPASFAVSLNGAK |  |  |  |  |  |  |
|  |  |  | 1533.78 | *AEAGVPAEFSIWTR |  |  |  |  |  |  |
|  |  |  | 1756.92 | VTYTPMAPGSYLISIK |  |  |  |  |  |  |
|  |  |  | 2018.03 | IPEISIQDMTAQVTSPSGK |  |  |  |  |  |  |
|  |  |  | 2057.94 | *THEAEIVEGENHTYCIR |  |  |  |  |  |  |
|  |  |  | 2296.15 | GAGSYTIMVLFADQATPTSPIR |  |  |  |  |  |  |
|  |  |  | 2304.15 | GQHVPGSPFQFTVGPLGEGGAHK |  |  |  |  |  |  |
|  |  |  | 2467.19 | *FNEEHIPDSPFVVPVASPSGDAR |  |  |  |  |  |  |
| 1670 | 4/6 | 68 | 927.50 | YLYEIAR | Serum albumin | ALBU_HUMAN | 43.21 ± 19.30 / 19.60 ± 7.30 | 55594 / 66472 | 5.72 / 5.67 | +2.20 |
|  |  |  | 984.52 | TYETTLEK |  |  |  |  |  |  |
|  |  |  | 1138.50 | CCTESLVNR |  |  |  |  |  |  |
|  |  |  | 1639.93 | *KVPQVSTPTLVEVSR |  |  |  |  |  |  |
| 1818 | 8/40 | 271 | 858.44 | AVPTDEAR | Ras-related protein Rab-11A | RB11A_HUMAN | 205.93 ± 135.18 / 337.57 ± 138.15 | 21171 / 24393 | 5.59 / 6.12 | -1.64 |
|  |  |  | 944.48 | *AITSAYYR |  |  |  |  |  |  |
|  |  |  | 1080.57 | *STIGVEFATR |  |  |  |  |  |  |
|  |  |  | 1160.57 | *HLTYENVER |  |  |  |  |  |  |
|  |  |  | 1274.62 | *AQIWDTAGQER |  |  |  |  |  |  |
|  |  |  | 1289.74 | GAVGALLVYDIAK |  |  |  |  |  |  |
|  |  |  | 1384.69 | *FTRNEFNLESK |  |  |  |  |  |  |
|  |  |  | 2040.06 | ELRDHADSNIVIMLVGNK |  |  |  |  |  |  |
| 1833 | 6/39 | 222 | 740.32 | EGSEYR | rho GDP-dissociation inhibitor 2 | GDIR2_HUMAN | 402.20 ± 303.02 / 617.94 ± 264.61 | 16888 / 22857 | 5.76 / 5.10 | -1.54 |
|  |  |  | 746.43 | TGVKVDK |  |  |  |  |  |  |
|  |  |  | 855.51 | *APNVVVTR |  |  |  |  |  |  |
|  |  |  | 966.48 | *YVQHTYR |  |  |  |  |  |  |
|  |  |  | 2688.38 | LTLVCESAPGPITMDLTGDLEALKK |  |  |  |  |  |  |
|  |  |  | 2960.42 | ATFMVGSYGPRPEEYEFLTPVEEAPK |  |  |  |  |  |  |
| 1836 | 6/11 | 132 | 762.39 | INVMNR | Integrin-linked protein kinase | ILK_HUMAN | 182.52 ± 156.69 / 311.17 ± 150.31 | 17663 / 51419 | 5.83 / 8.30 | -1.70 |
|  |  |  | 815.44 | *EGNAVAVR |  |  |  |  |  |  |
|  |  |  | 848.41 | MGQNLNR |  |  |  |  |  |  |
|  |  |  | 1180.61 | SAVVEMLIMR |  |  |  |  |  |  |
|  |  |  | 1333.72 | MGQNLNRIPYK |  |  |  |  |  |  |
|  |  |  | 1583.77 | *GDDTPLHLAASHGHR |  |  |  |  |  |  |
| 1856 | 6/4 | 104 | 1450.76 | AGNNMLLVGVHGPR | Filamin-A | FLNA_HUMAN | 489.08 ± 278.94 / 226.17 ± 96.95 | 120645 / 280608 | 5.97 / 5.70 | +2.16 |
|  |  |  | 1602.77 | *YNEQHVPGSPFTAR |  |  |  |  |  |  |
|  |  |  | 2440.21 | VSGQGLHEGHTFEPAEFIIDTR |  |  |  |  |  |  |
|  |  |  | 2249.24 | IVGPSGAAVPCKVEPGLGADNSVVR |  |  |  |  |  |  |
|  |  |  | 2467.20 | FNEEHIPDSPFVVPVASPSGDAR |  |  |  |  |  |  |
|  |  |  | 2893.56 | VGSAADIPINISETDLSLLTATVVPPSGR |  |  |  |  |  |  |
| 1913 | 8/23 | 318 | 880.44 | *HYELYR | Septin-11 | SEP11_HUMAN | 119.89 ± 91.17 / 245.19 ± 108.21 | 46341 / 49267 | 5.93 / 6.38 | -2.05^§^ |
|  |  |  | 1152.55 | *SLFNYHDTR |  |  |  |  |  |  |
|  |  |  | 1224.59 | *SYELQESNVR |  |  |  |  |  |  |
|  |  |  | 1233.66 | *RNEFLGELQK |  |  |  |  |  |  |
|  |  |  | 1555.72 | *FESDPATHNEPGVR |  |  |  |  |  |  |
|  |  |  | 1619.87 | *LTIVDTVGFGDQINK |  |  |  |  |  |  |
|  |  |  | 2070.96 | DTDPDSKPFSLQETYEAK |  |  |  |  |  |  |
|  |  |  | 2140.11 | NLSLSGHVGFDSLPDQLVNK |  |  |  |  |  |  |
| 1925 | 12/19 | 223 | 886.50 | DIELQIR | Dynamin-1-like protein | DNM1L_HUMAN | 91.12 ± 50.55 / 180.85 ± 140.70 | 52625 / 81877 | 5.93 / 6.37 | -1.98^§^ |
|  |  |  | 940.59 | *LGIIGVVNR |  |  |  |  |  |  |
|  |  |  | 1046.57 | SSVLESLVGR |  |  |  |  |  |  |
|  |  |  | 1171.56 | LYTDFDEIR |  |  |  |  |  |  |
|  |  |  | 1355.63 | YIETSELCGGAR |  |  |  |  |  |  |
|  |  |  | 1442.68 | *ICYIFHETFGR |  |  |  |  |  |  |
|  |  |  | 1637.89 | *LHDAIVEVVTCLLR |  |  |  |  |  |  |
|  |  |  | 1771.87 | SVTDSIRDEYAFLQK |  |  |  |  |  |  |
|  |  |  | 1774.99 | RPLILQLVHVSQEDK |  |  |  |  |  |  |
|  |  |  | 1861.91 | IIQHCSNYSTQELLR |  |  |  |  |  |  |
|  |  |  | 1871.80 | LDLMDAGTDAMDVLMGR |  |  |  |  |  |  |
|  |  |  | 2300.08 | LYTDFDEIRQEIENETER |  |  |  |  |  |  |
| 1935 | 12/22 | 65 | 848.46 | VADFGLAR | Proto-oncogene tyrosine-protein kinase Src | SRC_HUMAN | 88.86 ± 64.73 / 139.17 ± 48.19 | 47358 / 59835 | 6.00 / 7.10 | -1.57 |
|  |  |  | 987.51 | EVLDQVER |  |  |  |  |  |  |
|  |  |  | 1039.59 | LLLNAENPR |  |  |  |  |  |  |
|  |  |  | 1048.53 | VPYPGMVNR |  |  |  |  |  |  |
|  |  |  | 1215.60 | LDSGGFYITSR |  |  |  |  |  |  |
|  |  |  | 1223.59 | LIEDNEYTAR |  |  |  |  |  |  |
|  |  |  | 1234.63 | WTAPEAALYGR |  |  |  |  |  |  |
|  |  |  | 1261.65 | GRVPYPGMVNR |  |  |  |  |  |  |
|  |  |  | 1343.70 | KLDSGGFYITSR |  |  |  |  |  |  |
|  |  |  | 1546.70 | GAYCLSVSDFDNAK |  |  |  |  |  |  |
|  |  |  | 1789.91 | TQFNSLQQLVAYYSK |  |  |  |  |  |  |
|  |  |  | 2195.11 | LPQLVDMAAQIASGMAYVER |  |  |  |  |  |  |
| 2062 | 3/12 | 189 | 1930.07 | *LGLVFDDVVGIVEIINSK | Adenylyl cyclase-associated protein 1 | CAP1_HUMAN | 114.93 ± 83.97 / 198.74 ± 109.34 | 20556 / 51724 | 5.94 / 8.29 | -1.73 |
|  |  |  | 2073.02 | *VENQENVSNLVIEDTELK |  |  |  |  |  |  |
|  |  |  | 2827.32 | *SSEMNVLIPTEGGDFNEFPVPEQFK |  |  |  |  |  |  |
| 2076 | 6/10 | 129 | 762.39 | INVMNR | Integrin-linked protein kinase | ILK_HUMAN | 186.12 ± 178.27 / 356.77 ± 215.67 | 18125 / 51419 | 6.02 / 8.30 | -1.92 |
|  |  |  | 815.46 | ELLRER |  |  |  |  |  |  |
|  |  |  | 848.41 | MGQNLNR |  |  |  |  |  |  |
|  |  |  | 967.61 | APLRELLR |  |  |  |  |  |  |
|  |  |  | 1180.61 | SAVVEMLIMR |  |  |  |  |  |  |
|  |  |  | 1583.77 | *GDDTPLHLAASHGHR |  |  |  |  |  |  |
| 2337 | 2/3 | 95 | 927.49 | YLYEIAR | Serum albumin | ALBU_HUMAN | 18.71 ± 10.13 / 30.70 ± 15.85 | 33131 / 66472 | 6.12 / 5.67 | -1.64 |
|  |  |  | 1639.93 | KVPQVSTPTLVEVSR |  |  |  |  |  |  |
| 2818 | 14 | 105 | 701.41 | QERLR | Caldesmon | CALD1_HUMAN | 47.68 ± 30.18 / 21.18 ± 10.14 | 82480 / 93250 | 5.58 / 5.63 | +2.25 |
|  |  |  | 721.38 | AEFLNK |  |  |  |  |  |  |
|  |  |  | 815.47 | LEELRR |  |  |  |  |  |  |
|  |  |  | 1339.65 | LEQYTSAIEGTK |  |  |  |  |  |  |
|  |  |  | 1358.66 | HTENTFSRPGGR |  |  |  |  |  |  |
|  |  |  | 1581.79 | TTESQEETVVMSLK |  |  |  |  |  |  |
|  |  |  | 1991.07 | SAKPTKPAASDLPVPAEGVR |  |  |  |  |  |  |
|  |  |  | 2005.02 | QKEFDPTITDASLSLPSR |  |  |  |  |  |  |
|  |  |  | 2097.96 | TTTTNTQVEGDDEAAFLER |  |  |  |  |  |  |

Peptides submitted to MS/MS, as an additional analysis that led to the same protein identification, are marked with an asterisk. All differential proteins have a p value lower than 0.05 except those marked with ^§^ which have a p<0.01. ^†^Number of matched peptides. ^‡^Coverage of full length protein by tryptic peptides
