# Supplementary material for: Proteins Involved in Platelet Signaling Are Differentially Regulated in Acute Coronary Syndrome: A Proteomic Study
Source: PLoS One. 2010 Oct 14;5(10):e13404. doi: 10.1371/journal.pone.0013404 (PMC2954807; doi:10.1371/journal.pone.0013404)
Supplement: Table S2 — List of proteins differentially regulated in NSTE-ACS patients' platelets at different times in comparison with stable patients (SCAD). (0.07 MB DOC) [file pone.0013404.s003.doc]

**Table S2. List of proteins differentially regulated in NSTE-ACS patients´ platelets at different times in comparison with stable patients (SCAD)**.

| **Protein** | **Uniprot Code** | **Spot** | **Fold change Day 0** | **Fold change 5 days** | **Fold change 6 months** |
| --- | --- | --- | --- | --- | --- |
| **Actin Cytoplasmatic-1** | ACTB_HUMAN | 925 | +1.71 | +1.72* | +1.59 |
| **Adenylyl cyclase-associated protein 1** | CAP1_HUMAN | 2062 | -1.73 | -3.34* | - |
| **F-actin-capping protein subunit beta** | CAPZB_HUMAN | 1083 | -2.04 | -3.08* | - |
| **Filamin-A** | FLNA_HUMAN | 1311 | -1.62 | -3.85* | - |
|  |  | 1322 | -2.86* | -4.31* | - |
|  |  | 1626 | -1.69* | -3.31* | - |
|  |  | 1856 | +2.16 | +2.70 | - |
| **FYN-binding protein (ADAP, SLAP-130)** | FYB_HUMAN | 1148 | -1.98 | -3.26* | - |
| **Integrin-linked protein kinase** | ILK_HUMAN | 1836 | -1.70 | -4.77* | - |
|  |  | 2076 | -1.92 | -3.47* | - |
| **Myosin-9** | MYH9_HUMAN | 885 | -1.86 | -1.72 | - |
| **Ras-related protein Rab-6B** | RAB6B_HUMAN | 1131 | -1.91 | -2.83* | - |
|  |  | 1504 | -1.54 | -2.50 | - |
| **Ras-related protein Rab-11A** | RB11A_HUMAN | 1501 | -1.57 | -2.89* | - |
|  |  | 1818 | -1.64 | -4.05* | - |
| **Rho GDP-dissociation inhibitor 2** | GDIR2_HUMAN | 1833 | -1.54 | -3.78* | -1.59 |
| **Septin-11** | SEP11_HUMAN | 1913 | -2.05 | -1.90 | - |
| **Serum Albumin** | ALBU_HUMAN | 1670 | +2.20 | +1.99* | - |
|  |  | 2337 | -1.64 | -1.91 | - |
| **Talin-1** | TLN1_HUMAN | 414 | -1.97 | -1.69* | -2.32* |
|  |  | 798 | -2.15* | -2.63* | - |
|  |  | 905 | -2.48* | -2.44 | - |
|  |  | 1169 | -1.67* | -1.96* | - |
|  |  | 1184 | +3.36* | +1.99 | +1.88 |
|  |  | 1186 | -1.59* | -1.55 | - |
|  |  | 1254 | -2.07* | -1.66 | - |
|  |  | 1614 | +2.60* | +3.56 | - |
| **Tropomyosin alpha chain 3** | TPM3_HUMAN | 299 | -3.90* | -2.08 | -5.90 |

Differentially regulated protein features not present in this table were only significant on admission. All differentially regulated protein spots have a p<0.05 except those highlighted with an asterisk, which have a p<0.01.
