# Supplementary material for: Proteins Involved in Platelet Signaling Are Differentially Regulated in Acute Coronary Syndrome: A Proteomic Study
Source: PLoS One. 2010 Oct 14;5(10):e13404. doi: 10.1371/journal.pone.0013404 (PMC2954807; doi:10.1371/journal.pone.0013404)
Supplement: Supporting Information S1 — (0.03 MB DOC) [file pone.0013404.s004.doc]

**Supplementary Methods**

**Surface expression of platelet αIIbβ3**

Platelet-rich plasma (PRP) was obtained as described previously [García A, 2007]. The determination of the platelet surface expression of αIIbβ3 integrin was performed by flow cytometry by using 70 µl aliquots of PRP. PRP aliquots were incubated for 30 min at room temperature in the dark with mouse monoclonal antibodies anti-human CD41 (integrin αIIb) FITC-conjugated (Dako, Denmark), and anti-human CD61 PE-conjugated (BD Biosciences, CA, USA). Isotype controls were from BD Biosciences. The aliquots were washed twice with FACSFlow (BD Biosciences) and finally resuspended in the same fluid. Surface fluorescence was analyzed in a flow cytometer (FACScalibur, BD Biosciences) and mean fluorescence intensity (MFI) was used as the index of receptor expression. Data was analysed using Cellquest software from BD Biosciences.

**Two-dimensional gel electrophoresis**

1. Proteomics:

Immobilized pH gradient (IPG) strips (4-7, 24 cm, GE Healthcare (Uppsala, Sweden)) were rehydrated in the sample, and isoelectric focusing (IEF) was in a Multiphor (GE Healthcare) for 85 Kvh at 17^o^C. Following focusing, the IPG strips were immediately equilibrated for 15 min in 4 M urea, 2 M thiourea, 130 mM DTT, 50 mM Tris pH 6.8, 2% w/v SDS, 30% v/v glycerol. The IPG strips were placed on top of the second dimension gels and embedded with 0.5% melted agarose. Proteins were separated in the second dimension by SDS-polyacrylamide gel electrophoresis (PAGE) on 10% gels strengthened with Rhinohide^TM^ (Invitrogen), as described in the manufacturer’s manual, at run conditions of 10^o^C, 20 mA per gel for 1 h, followed by 40 mA per gel for 4 h by using an Ettan Dalt 6 system (GE Healthcare).

1. Western blotting

IPG strips were 4-7, 7 cm (GE Healthcare). IEF (first dimension) was for 11Kvh; second dimension was on 4-12% Bis-Tris gels (Invitrogen), using MOPS (Invitrogen) as running buffer. Following the electrophoresis, proteins were transferred onto PVDF membranes. Immunodetection was conducted as indicated below.

**Western blotting**

For 1D western blotting, 40 μg of protein were electrophoresed on 10% SDS-polyacrylamide gels and electrotransferred onto polyvinylidene fluoride (PVDF) membranes (GE Healthcare). The membranes were blocked in 5% BSA in TBS-T (20 mM Tris-HCl (pH 7.6), 150 mM NaCl and 0.1% Tween 20) overnight at 4^o^C and incubated for 90 min at room temperature with the following primary antibodies: mouse anti-ILK (1:200), rabbit anti-Src (1:200) and rabbit anti-SPARC (1:500) - all from Santa Cruz Biotechnology, Inc. (Delaware, CA, USA) - and mouse anti-β-actin (1:1000) from Millipore (Temecula, CA, USA). Following washes in TBS-T, the blots were exposed to horseradish peroxidase-labelled goat anti-rabbit or goat anti-mouse antibodies (dilution 1:2000) (Pierce, Rockford, USA) for 1 hour. Membranes were washed again and processed using an enhanced-chemiluminiscence system (ECL, Pierce, Rockford, USA) and quantified by densitometry.

For 2D western blotting, 100 μg of protein were resuspended in 2D sample buffer. 2-DE was performed as indicated above. Following the electrophoresis, proteins were transferred onto PVDF membranes. Immunodetection was conducted as indicated above.

**References**

García, A. Two-dimensional gel electrophoresis in platelet proteomics research. *Methods Mol. Med.* **2007**, *139*, 339-353.
