# Supplementary material for: Proteins Involved in Platelet Signaling Are Differentially Regulated in Acute Coronary Syndrome: A Proteomic Study
Source: PLoS One. 2010 Oct 14;5(10):e13404. doi: 10.1371/journal.pone.0013404 (PMC2954807; doi:10.1371/journal.pone.0013404)
Supplement: Supporting Information S2 — (2.99 MB PDF) [file pone.0013404.s005.pdf]

Experiment overview

Experiment	SCASEST
Comparison	NSTE_ACS vs SCAD
Groups	NSTE_ACS, SCAD

Experiment statistics

Statistical test	Anova & Mann-W
Applied filters	Only 'star rated' spots are included
Number filtered spots	40

Group color legend

NSTE_ACS   SCAD

Protein overview

Overview gel name: ACS15

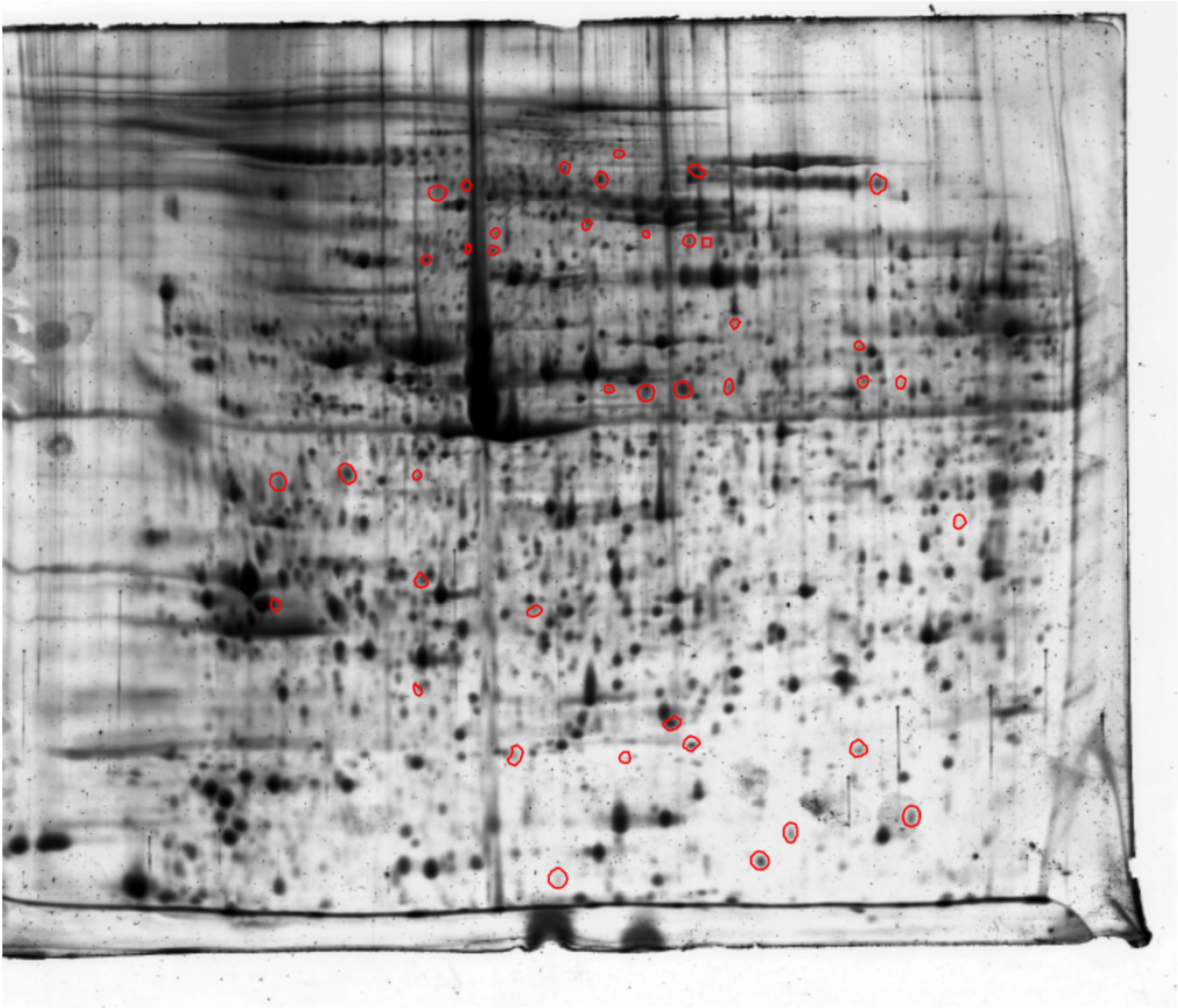

Filtered spots

ID 225
Anova 8.405e-4
Mann-W 7.404e-4
Presence 100 %
Fold 2.13
Volume 318

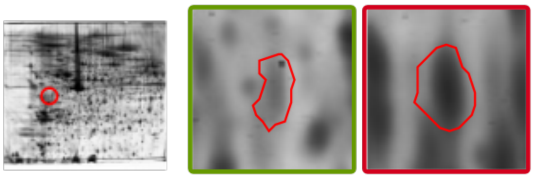

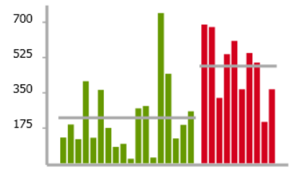

SPARC (Secreted protein acidic and rich in cysteine)

ID 299
Anova 4.253e-4
Mann-W 7.288e-4
Presence 96 %
Fold 3.90
Volume 1191

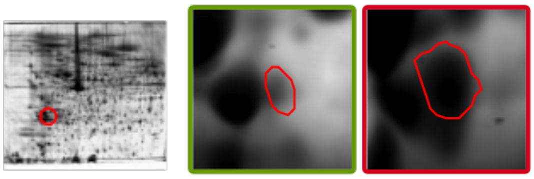

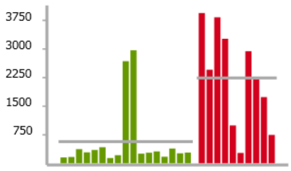

Tropomyosin alpha chain 3

ID 414
Anova 0.0100
Mann-W 0.0203
Presence 67 %
Fold 1.97
Volume 152

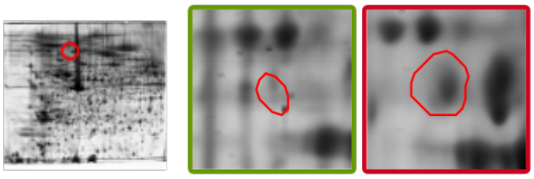

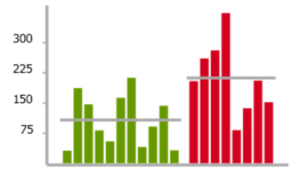

Talin-1

ID 450
Anova 0.0258
Mann-W 0.0208
Presence 100 %
Fold 1.63
Volume 117

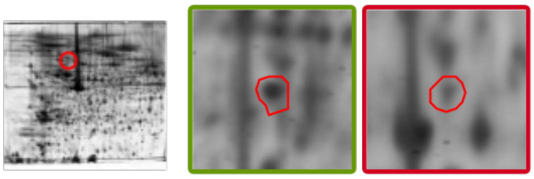

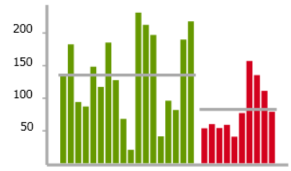

Alpha-actinin-1

ID 602
Anova 0.0236
Mann-W 0.0052
Presence 100 %
Fold 1.93
Volume 298

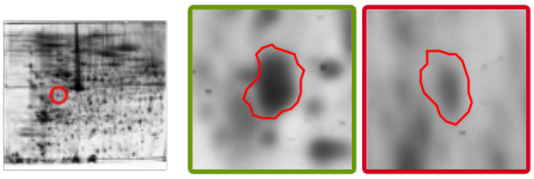

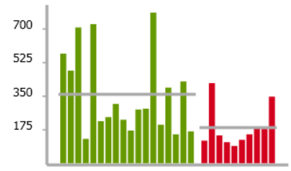

Zyxin

ID 639
Anova 0.0270
Mann-W 0.0185
Presence 85 %
Fold 2.26
Volume 96

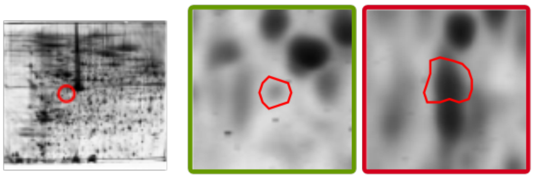

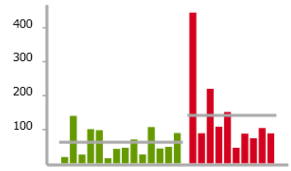

Talin-1

ID 653
Anova 0.0013
Mann-W 0.0018
Presence 92 %
Fold 2.06
Volume 137

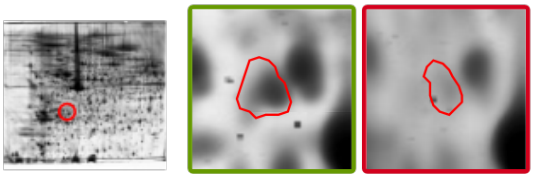

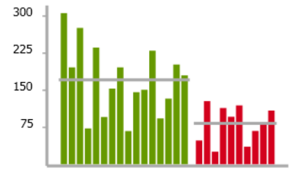

MAPRE-1 (Microtubule-associated protein RP/EB family member 1)

ID 713
Anova 0.0182
Mann-W 0.0196
Presence 89 %
Fold 1.66
Volume 167

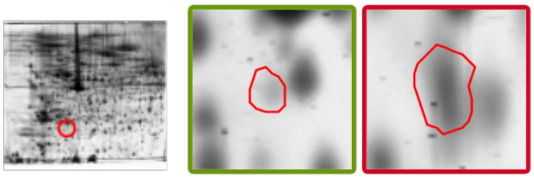

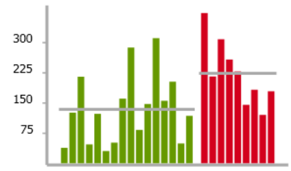

Ras-related protein Rab-27B

ID 798
Anova 1.789e-4
Mann-W 4.696e-4
Presence 100 %
Fold 2.15
Volume 627

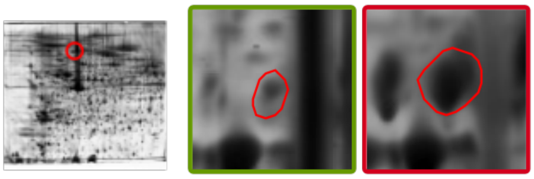

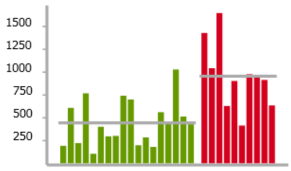

Talin-1

ID	885
Anova	0.0258
Mann-W	0.0103
Presence	92 %
Fold	1.86
Volume	158

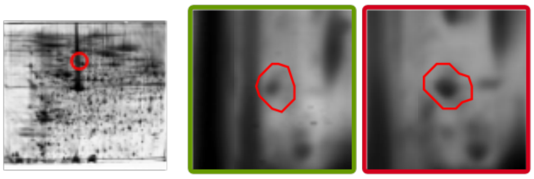

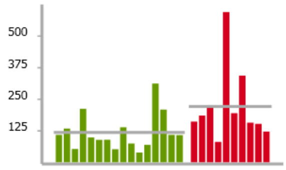

Myosin-9

ID	905
Anova	0.0028
Mann-W	0.0014
Presence	92 %
Fold	2.48
Volume	105

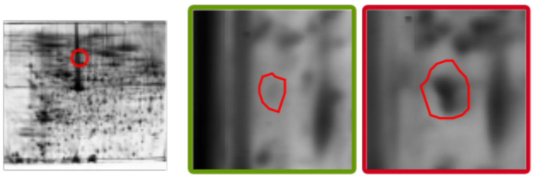

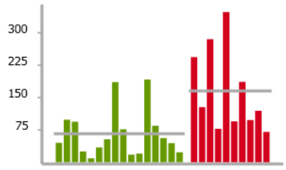

Talin-1

ID	925
Anova	0.0733
Mann-W	0.0308
Presence	96 %
Fold	1.71
Volume	117

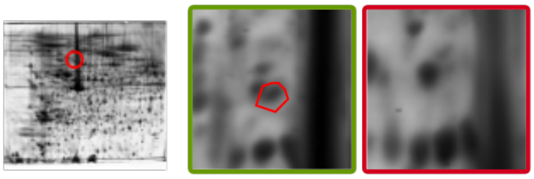

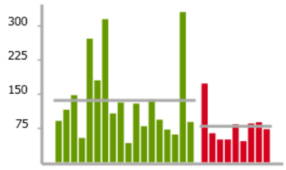

Actin Cytoplasmatic-1

ID	1083
Anova	0.0121
Mann-W	0.0122
Presence	92 %
Fold	2.04
Volume	186

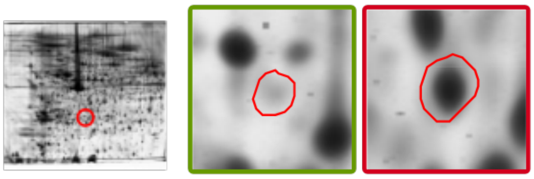

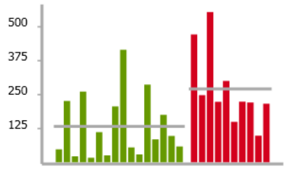

F-actin-capping protein subunit beta

ID	1131
Anova	0.0041
Mann-W	0.0015
Presence	92 %
Fold	1.91
Volume	73

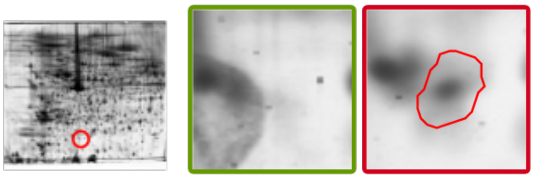

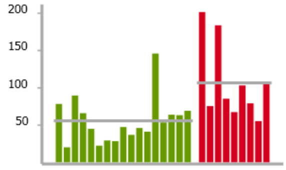

Ras-related protein Rab-6B

ID	1148
Anova	0.0069
Mann-W	0.0124
Presence	71 %
Fold	1.98
Volume	123

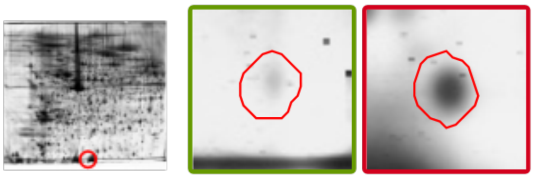

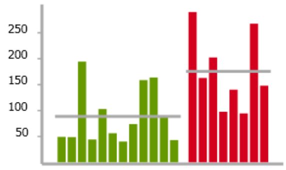

FYN-binding protein (ADAP, SLAP-130)

ID	1169
Anova	8.280e-4
Mann-W	0.0021
Presence	100 %
Fold	1.67
Volume	549

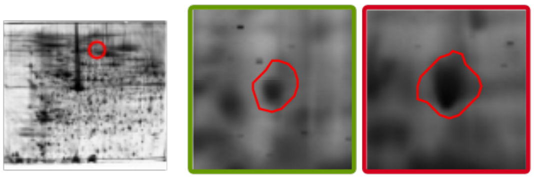

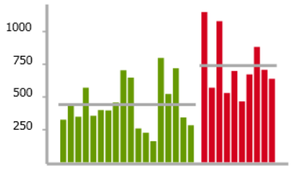

Talin-1

ID	1184
Anova	0.0022
Mann-W	0.0012
Presence	78 %
Fold	3.36
Volume	48

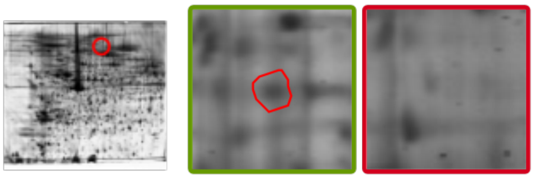

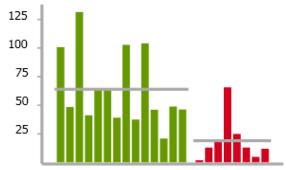

Talin-1

ID	1186
Anova	0.0069
Mann-W	0.0014
Presence	92 %
Fold	1.59
Volume	180

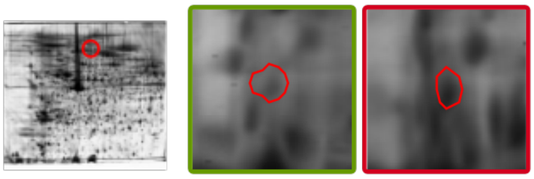

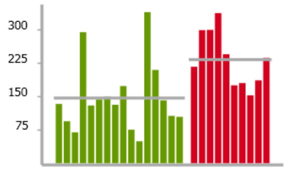

Talin-1

ID	1254
Anova	0.0269
Mann-W	0.0089
Presence	78 %
Fold	2.07
Volume	162

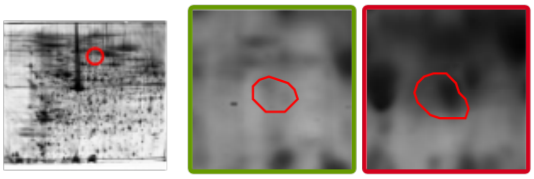
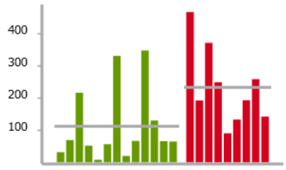

Talin-1

ID	1284
Anova	0.0127
Mann-W	0.0110
Presence	96 %
Fold	1.85
Volume	31

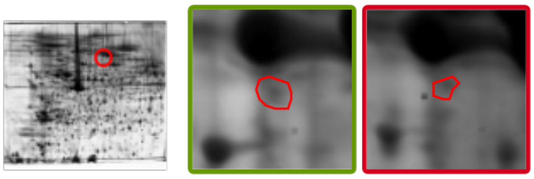
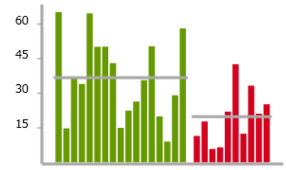

Serum Albumin

ID	1311
Anova	0.0379
Mann-W	0.0156
Presence	100 %
Fold	1.62
Volume	809

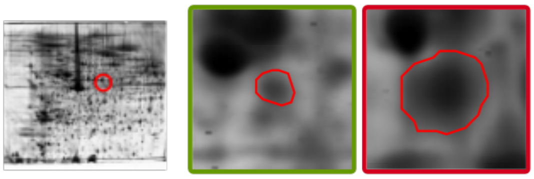
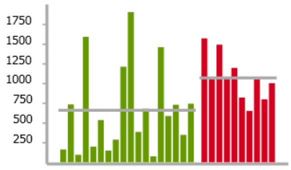

Filamin-A

ID	1322
Anova	8.021e-4
Mann-W	7.404e-4
Presence	100 %
Fold	2.86
Volume	206

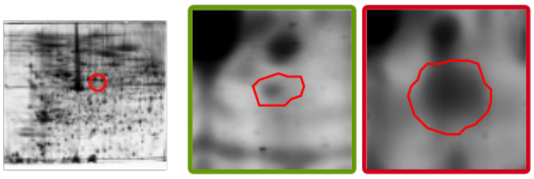
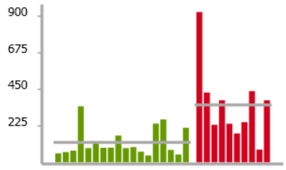

Filamin-A

ID	1501
Anova	0.0036
Mann-W	0.0035
Presence	96 %
Fold	1.57
Volume	584

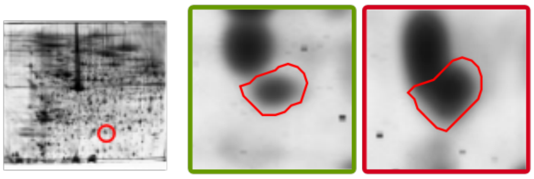
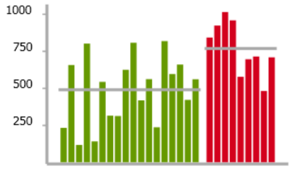

Ras-related protein Rab-11A

ID	1504
Anova	0.0523
Mann-W	0.0460
Presence	96 %
Fold	1.54
Volume	57

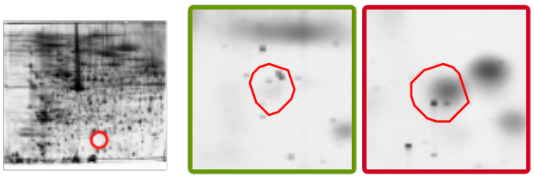
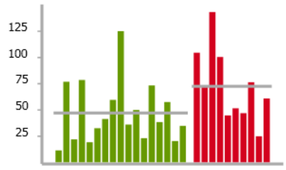

Ras-related protein Rab-6B

ID	1557
Anova	2.304e-4
Mann-W	7.404e-4
Presence	100 %
Fold	1.70
Volume	971

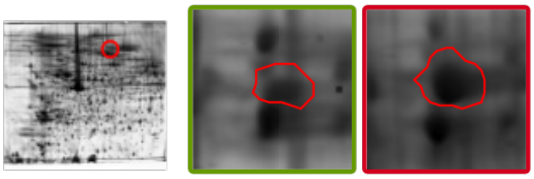
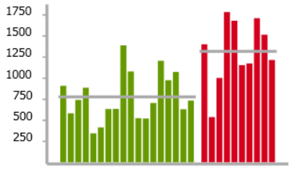

Talin-1

ID	1614
Anova	0.0493
Mann-W	0.0033
Presence	78 %
Fold	2.60
Volume	94

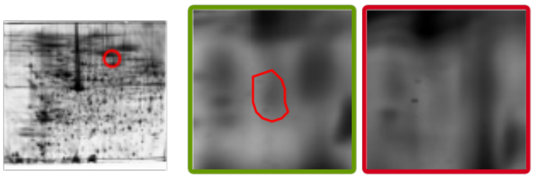
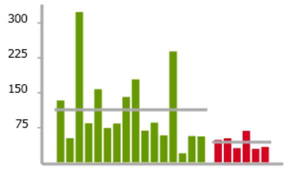

Talin-1

ID	1626
Anova	0.0049
Mann-W	0.0028
Presence	92 %
Fold	1.69
Volume	1876

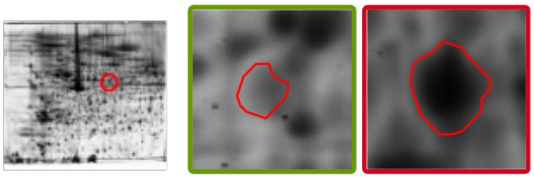
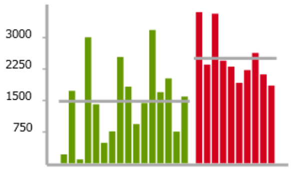

Filamin-A

ID	1631
Anova	3.471e-4
Mann-W	9.208e-4
Presence	100 %
Fold	2.02
Volume	287

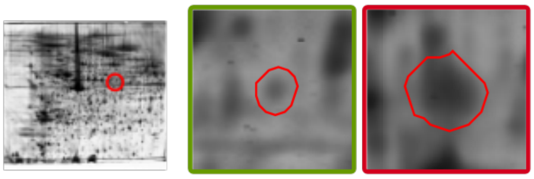
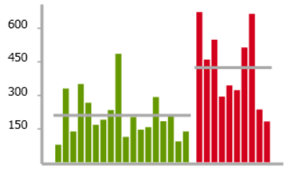

Filamin-A

ID	1670
Anova	0.0169
Mann-W	0.0107
Presence	71 %
Fold	2.20
Volume	37

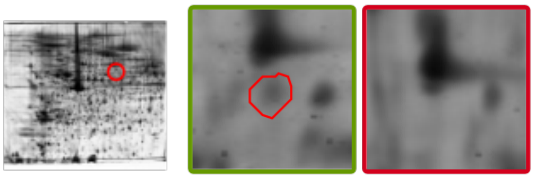
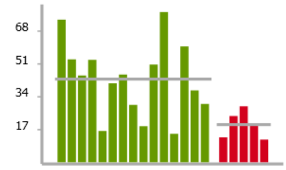

Serum Albumin

ID	1818
Anova	0.0213
Mann-W	0.0156
Presence	100 %
Fold	1.64
Volume	253

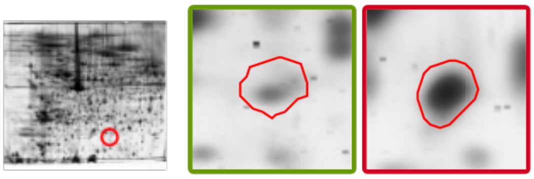
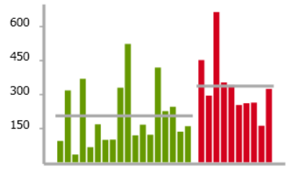

Ras-related protein Rab-11A

ID	1833
Anova	0.0766
Mann-W	0.0169
Presence	92 %
Fold	1.54
Volume	485

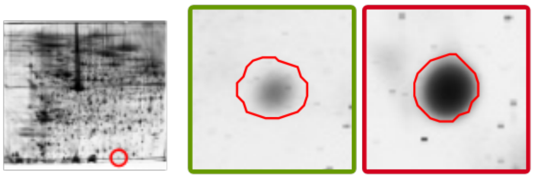
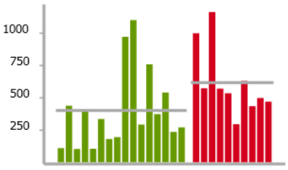

Rho GDP-dissociation inhibitor 2

ID	1836
Anova	0.0469
Mann-W	0.0521
Presence	96 %
Fold	1.70
Volume	230

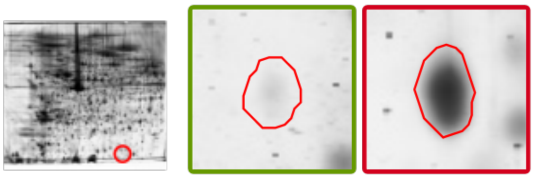
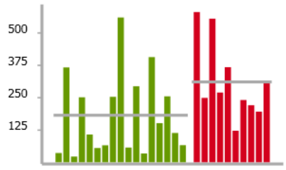

Integrin-linked protein kinase

ID	1856
Anova	0.0128
Mann-W	0.0101
Presence	85 %
Fold	2.16
Volume	390

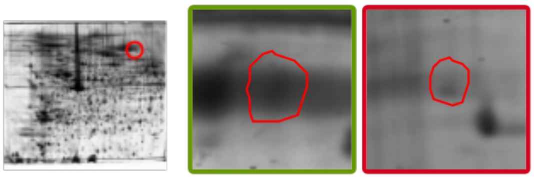
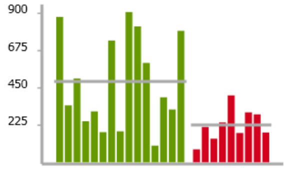

Filamin-A

ID	1913
Anova	0.0031
Mann-W	9.208e-4
Presence	100 %
Fold	2.05
Volume	165

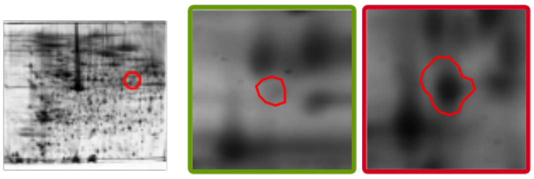
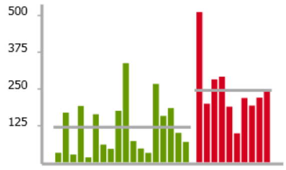

Septin-11

ID	1925
Anova	0.0377
Mann-W	0.0089
Presence	85 %
Fold	1.98
Volume	129

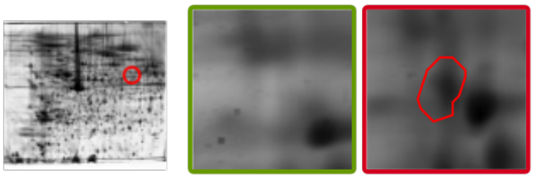
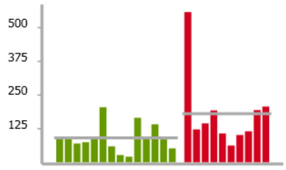

Dynamin-1-like protein

ID	1935
Anova	0.0590
Mann-W	0.0130
Presence	82 %
Fold	1.57
Volume	109

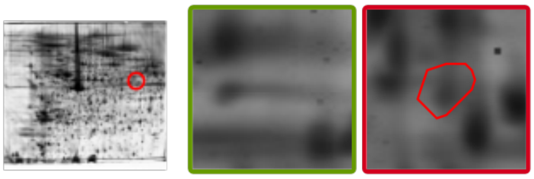
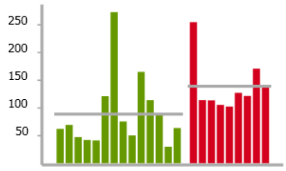

Proto-oncogene tyrosine-protein kinase Src

ID	2062
Anova	0.0316
Mann-W	0.0238
Presence	100 %
Fold	1.73
Volume	145

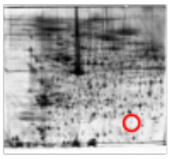

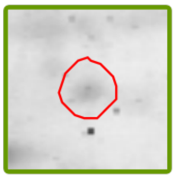

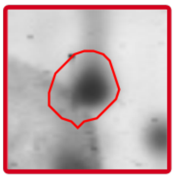

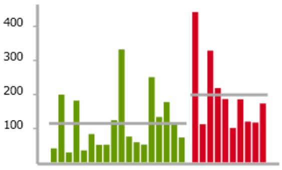

Adenylyl cyclase-associated protein 1

ID	2076
Anova	0.0383
Mann-W	0.0144
Presence	92 %
Fold	1.92
Volume	252

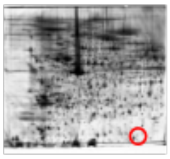

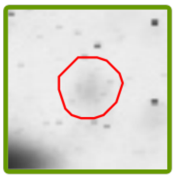

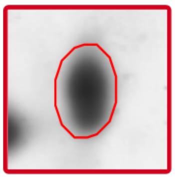

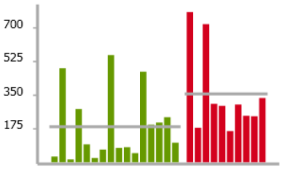

Integrin-linked protein kinase

ID	2337
Anova	0.0269
Mann-W	0.0250
Presence	92 %
Fold	1.64
Volume	23

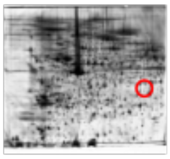

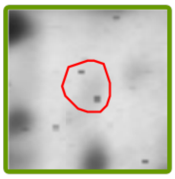

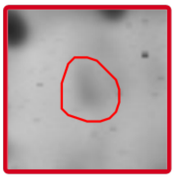

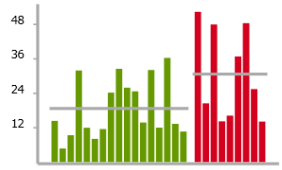

Serum Albumin

ID	2818
Anova	0.0279
Mann-W	0.0246
Presence	75 %
Fold	2.25
Volume	38

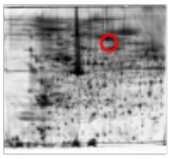

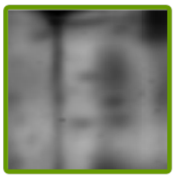

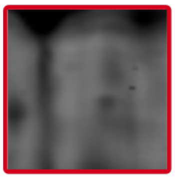

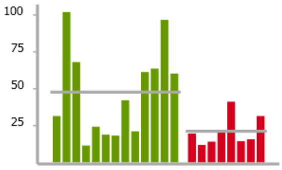

Caldesmon
